# Lower myostatin and higher MUC1 levels are associated with better response to mepolizumab and omalizumab in asthma: a protein–protein interaction analyses

**DOI:** 10.1186/s12931-023-02620-1

**Published:** 2023-12-06

**Authors:** Ayobami Akenroye, Tanawin Nopsopon, Laura Cho, Matthew Moll, Scott T. Weiss

**Affiliations:** 1 Division of Allergy and Clinical Immunology, Brigham and Women’s Hospital, 60 Fenwood Road, Boston, MA 02115 USA; 2 Channing Division of Network Medicine, Brigham and Women’s Hospital, 60 Fenwood Road, BostonBoston, MA 02115 USA; 3 Division of Pulmonary and Critical Care Medicine, Brigham and Women’s Hospital, 60 Fenwood Road, Boston, MA 02115 USA

**Keywords:** Asthma, Mepolizumab, Omalizumab, Proteomics, Network medicine, Protein–protein interaction, Monoclonal antibodies, Systems biology, Cytokines

## Abstract

**Introduction:**

Biomarkers are needed to inform the choice of biologic therapy in patients with asthma given the increasing number of biologics**.** We aimed to identify proteins associated with response to omalizumab and mepolizumab.

**Methods:**

Aptamer-based proteomic profiling (SomaScan) was used to assess 1437 proteins from 51 patients with moderate to severe asthma who received omalizumab (n = 29) or mepolizumab (n = 22). Response was defined as the change in asthma-related exacerbations in the 12 months following therapy initiation. All models were adjusted for age, sex, and pre-treatment exacerbation rate. Additionally, body mass index was included in the omalizumab model and eosinophil count in the mepolizumab model. We evaluated the association between molecular signatures and response using negative binomial regression correcting for the false discovery rate (FDR) and gene set enrichment analyses (GSEA) to identify associated pathways.

**Results:**

Over two-thirds of patients were female. The average age for omalizumab patients was 42 years and 57 years for mepolizumab. At baseline, the average exacerbation rate was 1.5/year for omalizumab and 2.4/year for mepolizumab. Lower levels of LOXL2 (unadjusted p: 1.93 × 10E−05, FDR-corrected: 0.028) and myostatin (unadjusted: 3.87 × 10E−05, FDR-corrected: 0.028) were associated with better response to mepolizumab. Higher levels of CD9 antigen (unadjusted: 5.30 × 10E−07, FDR-corrected: 0.0006) and MUC1 (unadjusted: 1.15 × 10E−06, FDR-corrected: 0.0006) were associated with better response to omalizumab, and LTB4R (unadjusted: 1.12 × 10E−06, FDR-corrected: 0.0006) with worse response. Protein–protein interaction network modeling showed an enrichment of the TNF- and NF-kB signaling pathways for patients treated with mepolizumab and multiple pathways involving MAPK, including the FcER1 pathway, for patients treated with omalizumab.

**Conclusions:**

This study provides novel fundamental data on proteins associated with response to mepolizumab or omalizumab in severe asthma and warrants further validation as potential biomarkers for therapy selection.

**Supplementary Information:**

The online version contains supplementary material available at 10.1186/s12931-023-02620-1.

## Key messages

1. Higher levels of LOXL2 and myostatin may correlate with worse response to mepolizumab
2. Higher levels of CD9 antigen and MUC1 may correlate with better response to omalizumab.
3. The CARD-BCL10-MALT1 (CBM) complex and MAPK may offer important clues into a single biomarker for both omalizumab and mepolizumab response.

**Supplementary Information:**

The online version contains supplementary material available at 10.1186/s12931-023-02620-1.

## Introduction

With a growing number of monoclonal antibodies (‘biologics’) approved for the treatment of moderate to severe asthma, patients and their providers are having to make an increasing number of therapeutic decisions on the optimal therapy for patients who are eligible for two or more of the six currently approved biologics. Given the high overlap in eligibility for these biologics [1], and the limited information on distinguishing biomarkers associated with treatment response [2, 3], research on biomarkers in this class of patients is sorely needed to optimize use of these expensive therapies.

Omalizumab, an anti-immunoglobulin E agent, and mepolizumab, an anti-interleukin-5 agent, were the two biologics first approved for the treatment of moderate-severe asthma and remain two of the most widely used biologics worldwide [4–7]. Patients with eosinophilic asthma, the clinical phenotype mepolizumab is approved for, also often have allergic disease, the phenotype targeted by omalizumab [1, 8]. Thus, identifying biomarkers associated with omalizumab and/or mepolizumab response will be helpful to clinicians as they seek to optimize treatment of their patient’s asthma.

Proteomics provides one such approach for identifying biomarkers associated with response to omalizumab and mepolizumab. A study of proteins and their interactions can provide a deeper understanding of the function of dynamic biologic systems beyond genomics which is further upstream and immutable [9, 10]. With the increasing availability of proteomics assays, serum protein biomarkers associated with response to omalizumab and mepolizumab may be helpful. Proteomic profiling of nasal samples, exhaled breath, airway epithelium, urine, and serum have been used to probe various asthma-related phenotypes. These include evaluating the role of T-helper cells in pediatric obese asthma [11], identifying proteins associated with airway inflammation [12, 13], association of inflammasome-related proteins with susceptibility to rhinovirus infections in patients with asthma [14], and plasma protein biomarkers of eosinophilic and neutrophilic asthma phenotypes [15]. While there are multiple ongoing consortia and projects to study multi-omics of asthma in general with an evolving focus on response to biologic therapies, such as the Severe Asthma Research Program (SARP) [16], the Unbiased Biomarkers Predictive of Respiratory Disease Outcomes (U-BIOPRED) [17], and the Precision Medicine Intervention in Severe Asthma (PRISM) study [18], there is a dearth of studies to identify protein biomarkers of response to any of the respiratory biologics, but prescriptions for these biologics continue to increase exponentially worldwide [3, 5].

The goal of this study was to identify proteins associated with response to omalizumab and mepolizumab in a single site cohort of patients with asthma. In addition, we sought to identify single protein biomarker(s) that might be helpful in identifying a patient likely to respond to omalizumab or mepolizumab, and to identify biological pathways associated with treatment response.

## Methods

### Study population and plasma samples

We obtained human plasma samples from 51 subjects with moderate to severe asthma from the Mass General Brigham (MGB) Biobank. The MGB IRB-approved Biobank has been previously described [19]. In brief, it includes samples from individuals seen within the MGB health care system who have volunteered or not opted out of their samples being collected in the biobank. Sample collection and storage follow standard biobanking protocols. These plasma samples were stored at − 80 °C with no prior freeze–thaw cycle. We identified 29 patients who initiated omalizumab and 22 who initiated mepolizumab for severe asthma and who had ethylenediaminetetraacetic acid (EDTA) plasma samples collected prior to the date of therapy initiation. To ensure patients initiated these biologics for control of their asthma, we excluded patients with other comorbidities which were alternate indications for these biologics.

### Proteomics assay

We performed relative quantification of 1437 proteins using the aptamer based SOMAScan assay. Based on our knowledge of asthma pathogenesis, the targets of omalizumab and mepolizumab, and prior studies, we preselected these 1437 proteins from a list of 7000 possible protein targets. These included proteins from a wide range of inflammatory pathways, cytokines, and chemokines. Assay details have been previously described [20, 21]. In brief, modified fluorescent single stranded DNA sequences called aptamers (SOMAmer reagents) bind to the protein targets in the biological sample. Subsequently, unbound proteins and nonspecifically bound SOMAmer reagents are removed in multiple washing steps. Protein concentrations are measured by hybridization of these SOMAmer reagents to complementary sequences on a DNA microarray and readouts done in relative fluorescent units (RFU). The RFU is directly proportional to each protein’s abundance within the specific sample. Details of Somalogic’s data standardization and quality control processes have also been previously described [20, 21]. This includes the addition of a pool of 11 matrix-matched, in this case EDTA Plasma, adult normal donor experimental controls to each 96-well plate to account for batch effects and buffer background levels of the assay. Data standardization is done using sample-specific signal normalization to overall signals within dilution bins and calibration to decrease between plate variability. Intraassay coefficients of variation based on RFU are ~ 5% suggesting good quality measures for the measured proteins.

### Defining clinical response: change in exacerbations

The primary outcome of interest was the change in asthma-related exacerbations in the one year following initiation of mepolizumab or omalizumab. We defined exacerbations, in line with the National Institutes of Health (NIH) and the Agency for Healthcare Research and Quality recommendations, as an emergency room visit or urgent care visit with a primary code for asthma for which a course of steroids for ≥ 3 days was prescribed, or an asthma-related hospitalization [22].

### Identifying proteins associated with response

Using a negative binomial model, we modeled the association between protein abundances and the change in exacerbations over one year of follow up. All models were adjusted for age, sex, and pre-treatment exacerbation rate. Additionally, body mass index was included in the omalizumab model given that weight is included in the determination of omalizumab’s dose and theoretically, the volume of distribution of omalizumab may be associated with response. Eligibility for omalizumab in adults includes an IgE level between 30 and 700 IU/ml [3]. In patients with IgE within this range, omalizumab has been shown to be effective regardless of IgE level [23]. Due to this specific range of IgE in omalizumab-eligible patients and our limited sample size, we did not adjust for IgE level. For mepolizumab, an anti-eosinophilic agent, we adjusted for the baseline eosinophil count. We adjusted for the false discovery rate (FDR) using the Benjamini–Hochberg method and adjusting for confounders as noted above. A false discovery rate-corrected (FDR-c) p < 0.05 was considered statistically significant and less than 0.20 was used in exploratory analyses. We also evaluated overlap between the significant proteins associated with mepolizumab and with omalizumab response. Finally, we compared the relationship between the baseline unadjusted levels (‘high’ level- above median; ‘low’ level- below median) of the proteins significantly associated with therapeutic response and the change in exacerbations over follow up to the relationship between common clinical features (body mass index, BMI ≥ 30 vs < 30 kg/m^2^) and biomarkers (eosinophil count ≥ 300 vs < 300 cells/microliter and IgE ≥ 100 vs < 100 IU/mL) and the change in exacerbations.

### Hierarchical clustering

Thereafter, unsupervised hierarchical clustering of the identified significant proteins was done based on their relative protein abundances in each sample. We used the *hclust* package with a Euclidean distance metric and Ward clustering method based on log2-transformed and row-scaled protein expression and created heat maps with the *pheatmap* package. All analyses were done using R version 4.3.0.

### Gene set enrichment analyses (GSEA)

GSEA was used to identify the top associated functional pathways using the normalized enrichment score (NES) and visualized in a network map (gene-concept-cnet plot) [24]. The *cnetplot* depicts the linkages of these proteins to biological concepts using the Gene Ontology (GO) terms. The gene set enrichment analysis (GSEA) was conducted for all measured proteins using the biological processes (BP) domain of GO terms. We used a less conservative FDR-corrected threshold (P < 0.20) to define statistical significance for GSEA using the pre-ranked covariate-adjusted betas from the negative binomial model as described above. The *clusterProfiler* package was used to conduct GSEA and *enrichplot* package to visualize the results.

### Protein–protein interaction (PPI) network construction and response module

Given extensive evidence that functionally active proteins interact with other proteins in a PPI network, we evaluated the functional and physical connections between the proteins associated with therapeutic response using the Search Tool for the Retrieval of Interacting Genes (STRING; http://string-db.org) (version 11.0) online database [25, 26]. The STRING database is a publicly available database for the exploration of known and predicted protein–protein interactions and includes about 70 million proteins from various organisms, including 20,000 human proteins [26]. We limited interactions between proteins to those with at least a score of high confidence (≥ 0.70). Clusters were generated using the Markov Cluster Algorithm (MCL) within STRING. Thereafter, we evaluated the enriched pathways in the PPI network to gain systems-level insights into proteomic changes associated with biologic response and evaluated if this enrichment of the mapped therapeutic response module was statistically significant. That is, if the proteins associated with treatment response demonstrate more interactions and connections than expected of a random protein module of the same size.

## Results

### Characteristics of the cohort and outcome over follow up

The average age for patients who initiated mepolizumab was 57 years, 68% of these individuals were female, median eosinophil count was 269 cells per mcl, and patients had 2.4 exacerbations in the year prior to therapy initiation. The average age of the omalizumab group was 42 years, 90% of the group identified as White, mean BMI was 28.2 kg/m^2^, and the individuals had 1.5 exacerbations on average in the year prior to initiation of omalizumab (Table 1). Over one year of follow-up, 10 (45.5%) of the 22 mepolizumab patients demonstrated some improvement in exacerbations over one year of follow up with 7 (70%) of these 10 patients having a halving or more of their baseline exacerbation rate in the one year on mepolizumab. The remaining twelve patients included six (27.3%) who had similar exacerbation rates over follow up as at baseline and six patients (27.3%) who had higher exacerbations over the first year of therapy. Over one year of follow-up, 11 (37.9%) of the 29 omalizumab patients had a reduction in their exacerbations over follow up with 8 (73%) of these 11 patients having a halving or more of their baseline exacerbation rate. Ten of the remaining 18 individuals had similar exacerbation rates over follow up as at baseline and 8 fared worse in the year on omalizumab.

**Table 1:** Baseline characteristics of participants

	Mepolizumab	Omalizumab
N	22	29
Age in years, mean (SD)	57.2 (11.6)	42.0 (15.3)
Female, n (%)	15 (68)	23 (79)
White race, n (%)	17 (77)	26 (90)
Current smoker, n (%)	1 (4.5)	0 (0.0)
Former smoker, n (%)	5 (22.7)	3 (10.3)
BMI, kg/m^2^; mean (SD)	30.5 (6.6)	28.2 (7.5)
Baseline annualized exacerbation rate, mean (SD)	2.4 (2.0)	1.5 (1.6)
Blood eosinophil counts, cells/µL; median [IQR]	269 [90–395]^a^	121 [78–247]
Immunoglobulin E (IgE), IU/µL; median [IQR]	213 [44–482]	92 [45–281]^b^
Allergic rhinitis, n (%)	19 (86)	29 (100)

BMI, Body mass index; IQR, interquartile range; SD, standard deviation

^a^All patients with eosinophil count of < 150 cells per mcl at baseline visit had counts above 150 at least once in the year prior to mepolizumab initiation

^b^Two patients who started omalizumab for asthma and met other criteria for omalizumab were missing baseline IgE

### Proteins associated with response: mepolizumab

**Fig. 1:**
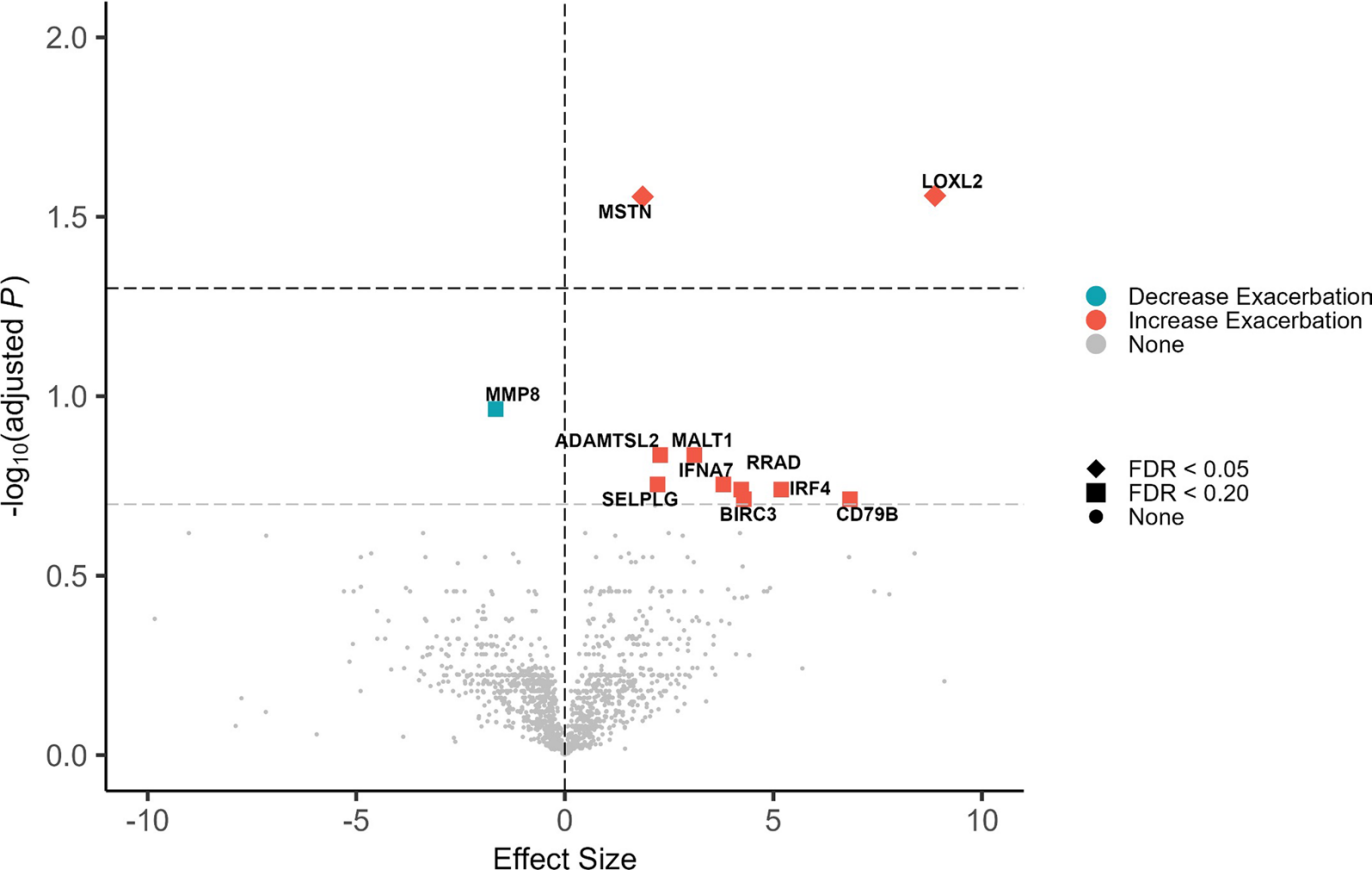
Proteins associated with response to mepolizumab. Modeled using negative binomial regression with post-mepolizumab exacerbation rate as a dependent variable and adjusted for age, sex, eosinophil counts, and pre-mepolizumab annualized exacerbation rate. Proteins with false discovery rate (FDR) < 0.20 were considered statistically significant. Diamonds indicate proteins that are significant at FDR < 0.05. Squares indicate proteins with significant association with FDR < 0.20. Circles (‘None’) indicate non-significant proteins. Effect sizes were presented as beta coefficients where positive (negative) values indicate association with increased (decreased) post-mepolizumab exacerbation. The vertical dashed lines represent no difference. The grey horizontal dashed line represents FDR < 0.20 and the black horizontal dashed line represents FDR < 0.05

**Fig. 2:**
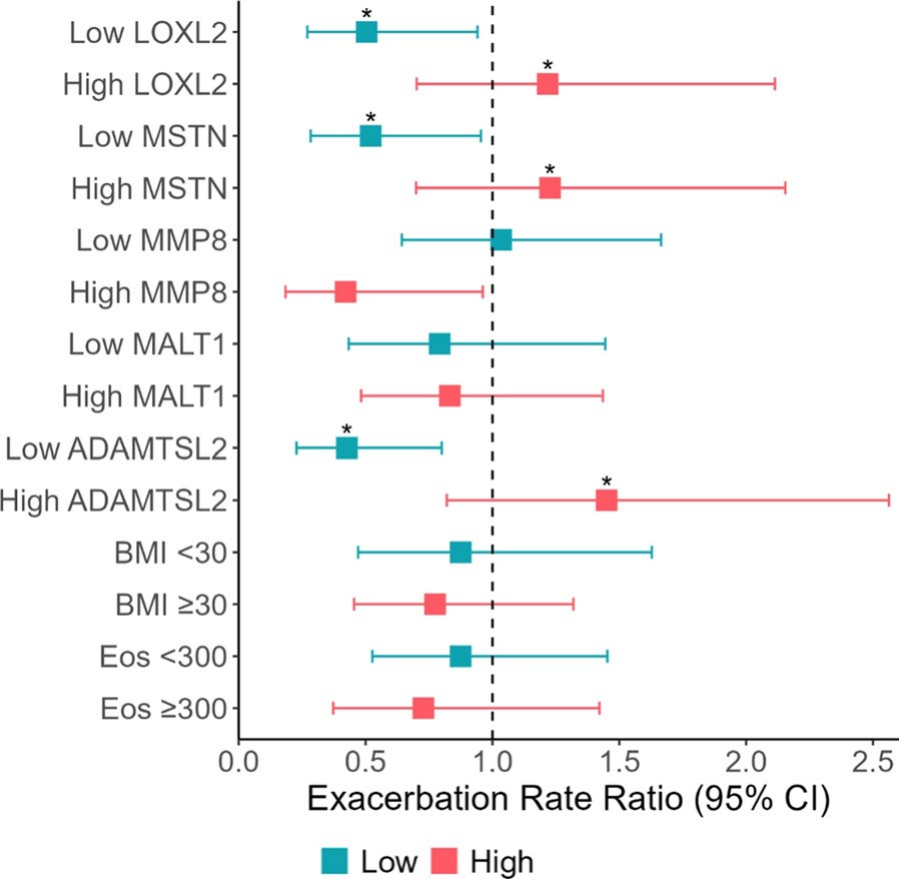
Forest plot showing the relationship between the exacerbation rate ratio and unadjusted levels of the top 5 proteins associated with mepolizumab response. ‘Low’ indicates protein level below the median and ‘high’ indicates levels at or above the median. Exacerbation rate ratios < 1.0 indicate better response to mepolizumab, that is greater reductions asthma exacerbation. *comparing groups, e.g., high vs low: p < 0.05. BMI, body mass index; CI, confidence interval; Eos, eosinophil counts

Lysyl oxidase like protein-2 (LOXL2; unadjusted p: 1.93 × 10E−05, FDR-corrected: 0.028) and myostatin (MSTN; also known as GDF-8- Growth derived factor-8; unadjusted: 3.87 × 10E−05, FDR-corrected: 0.028) were associated with increased risk of post-mepolizumab exacerbation (Fig. 1). Eleven proteins met significance at the FDR threshold of < 0.20 (Additional file 1: Table S1). This includes neutrophil collagenase (MMP-8), the only protein with high levels corresponding with lower risk, mucosa-associated lymphoid tissue lymphoma translocation protein 1 (MALT1), and interferon-related proteins (IFNA7 and IRF4) (Fig. 1 and Additional file 1: Fig. S1). The exacerbation rate was significantly lower in patients with low levels (below median) of LOXL2, myostatin, and ADAMTSL2. For MMP8, the exacerbation rate was higher in patients with low levels (Fig. 2). While patients with eosinophil counts of ≥ 300 cells per mcL was associated with greater reductions in exacerbations on mepolizumab, this did not reach statistical significance.

GSEA demonstrated 4 significantly enriched functional groups associated with response to mepolizumab (Additional file 1: Fig. S2). The strongest associations were with extracellular matrix organization and extracellular structure organization (both with NES 1.93, unadjusted p = 7.63 × 10E−05, FDR-corrected: 0.07, Additional file 1: Table S2). The gene-concept network map highlighted functional groups extracellular matrix, extracellular structure organization, and external encapsulating structure organization with leading edge interactions through LOXL2, ADAMTSL2, and CAV2 (Additional file 1: Fig. S3).

### PPI network of mepolizumab response module

**Fig. 3:**
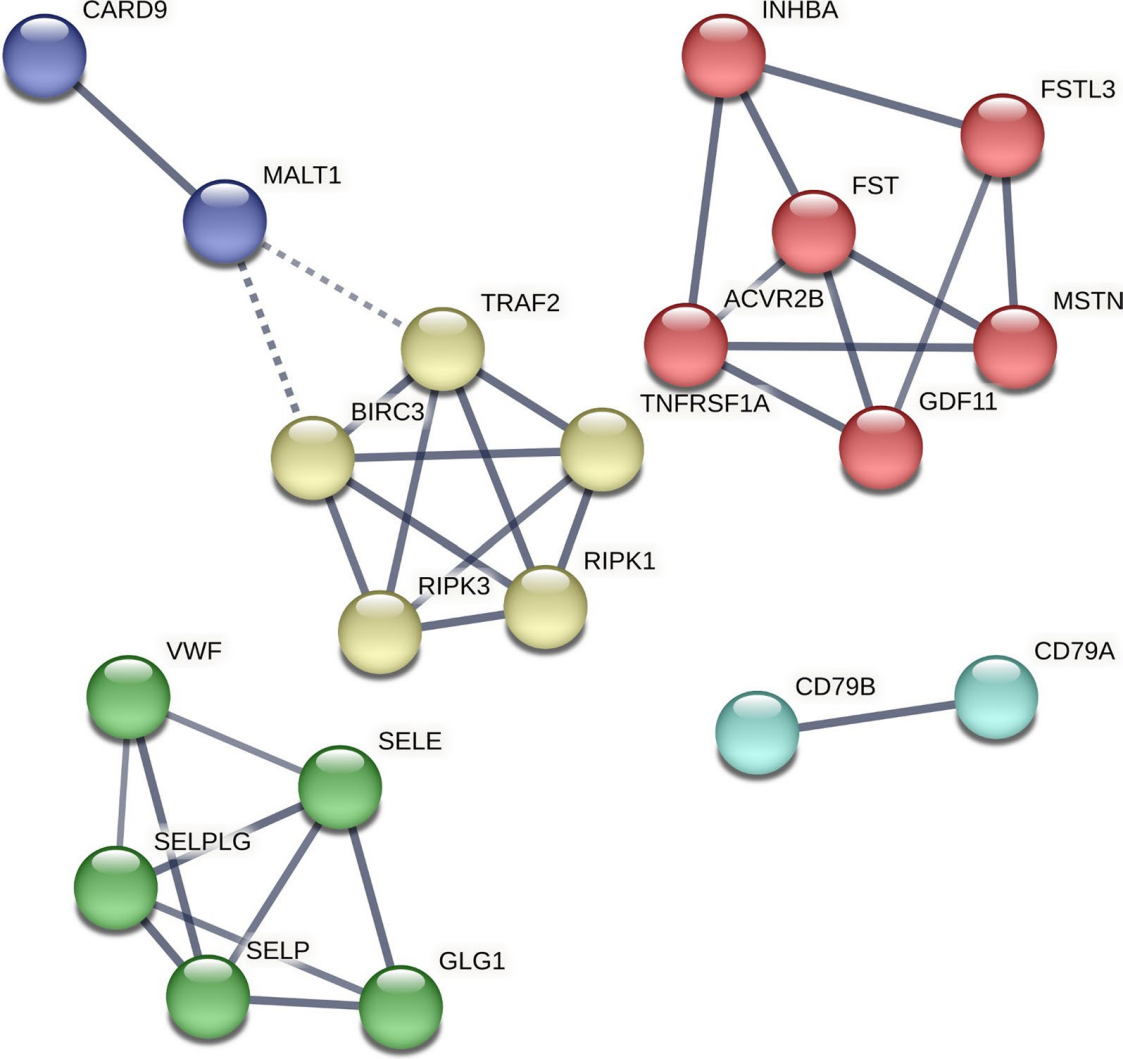
Protein–Protein Interaction (PPI) network of mepolizumab response. Network including interactions with at least a score of high confidence (≥ 0.70), 10 interactors in first shell, and 5 in the second shell. Clusters (color-coded) were generated using the Markov Cluster Algorithm (MCL) embedded in the STRING database

We evaluated the interactome of the eleven proteins associated with mepolizumab’s response (mepolizumab response module). Five (55%) of these proteins (IFNA7, IRF4, CD79B, and MALT1, and BIRC3) were directly connected without including other interactors (P for enrichment: < 0.001). Three of these five proteins (IRF4, CD79B, and MALT1) were associated with functional enrichment of the B-cell receptor signaling pathway (FDR p-value: 0.016). Including interactors in the network resulted in ten nodes and 33 edges, the MCL algorithm generated 5 unsupervised clusters (Fig. 3). Three of these clusters had 3 or more proteins in its network (Additional file 1: Table S3). The first cluster (PPI enrichment: < 1.0 × 10E−17) included myostatin and five other proteins involved in the activin and TGF-beta signaling pathways (enrichment strength: 2.03, FDR P: < 0.001) and in cytokine–cytokine receptor interaction (enrichment strength: 1.67, FDR P < 0.001). The second cluster was associated with enrichment in the TNF signaling pathway and included caspase-related proteins (RIPK1 and RIPK3). This cluster was related to cluster-5 which included CARD9 and MALT1. Cluster 4 included two proteins: CD79A and CD79B and was enriched for the B cell receptor signaling pathway (Additional file 1: Table S4).

### Proteins associated with response: omalizumab

**Fig. 4:**
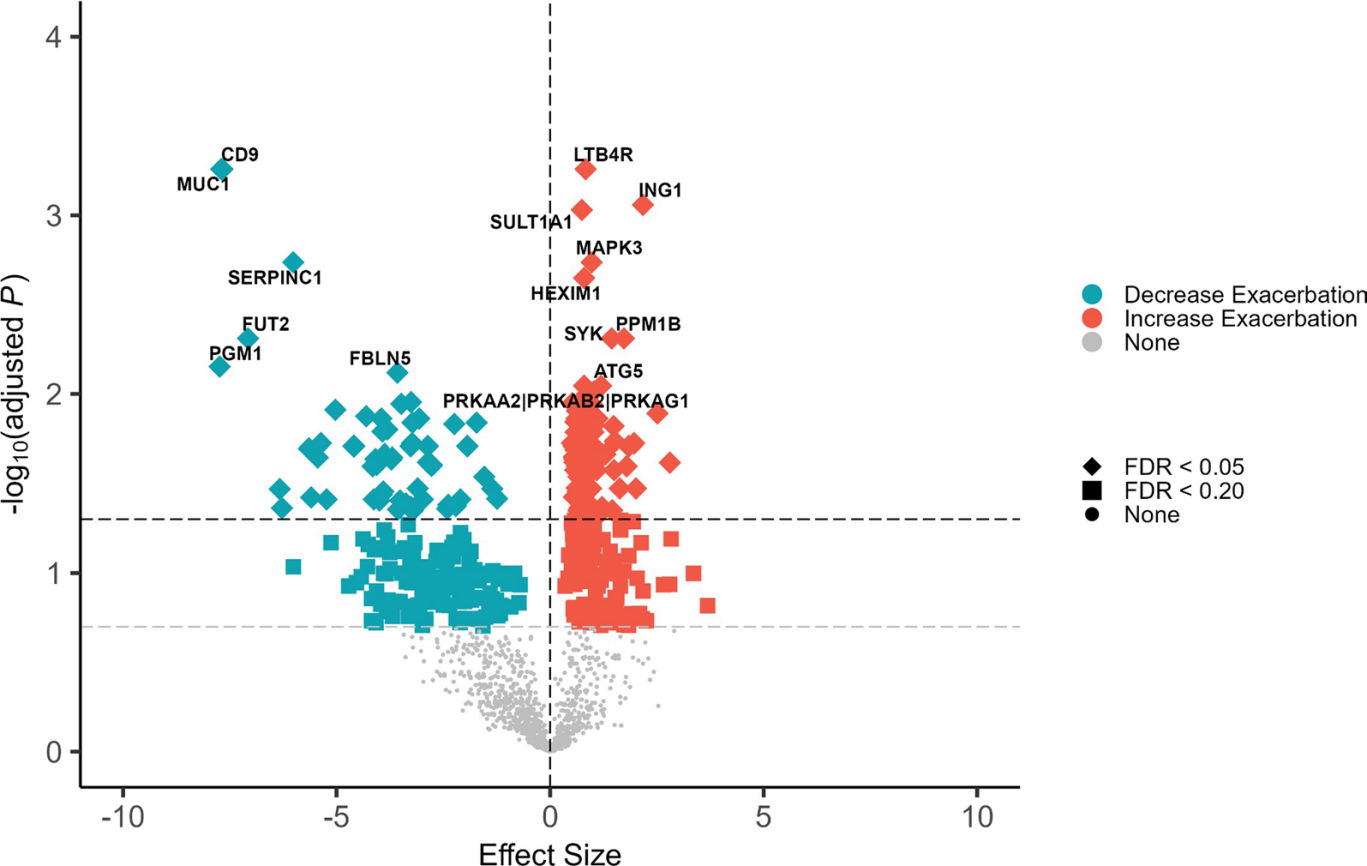
Proteins associated with response to omalizumab. Modeled using negative binomial regression with post-omalizumab exacerbation rate as a dependent variable and adjusted for age, sex, body mass index, and pre-omalizumab annualized exacerbation rate. Proteins with false discovery rate (FDR) < 0.20 were considered statistically significant. Diamonds indicate proteins that are significant at FDR < 0.05. Squares indicate proteins with significant association with FDR < 0.20. Circles (‘None’) indicate non-significant proteins. Effect sizes were presented as beta coefficients where positive (negative) values indicate association with increased (decreased) post-omalizumab exacerbation. The vertical dashed lines represent no difference. The grey horizontal dashed line represents FDR < 0.20 and the black horizontal dashed line represents FDR < 0.05

For omalizumab, 172 proteins met significance at the FDR-corrected threshold of P < 0.05 and 5 of these proteins at the P < 0.001 threshold. Higher levels of CD9 antigen (unadjusted: 5.30 × 10E−07, FDR-corrected: 0.0006) and mucin-1: region 3 (MUC1; unadjusted: 1.15 × 10E−06, FDR-corrected: 0.0006) were associated with better response to omalizumab, while higher levels of leukotriene B4 receptor 1 (LTB4R; unadjusted: 1.12 × 10E−06, FDR-corrected: 0.0006), inhibitor of growth protein 1 (ING1; unadjusted: 2.43 × 10E−06, FDR-corrected: 0.0008), and the sulfotransferase family 1A, SULT1A1 (unadjusted: 3.24 × 10E−06, FDR-corrected: 0.0009) were associated with worse response to omalizumab (Fig. 4 and Additional file 1: Table S5). Clustering of the top 80 proteins revealed some clustering by change in exacerbations but with no distinct cut-offs (Additional file 1: Fig. S4).

**Fig. 5:**
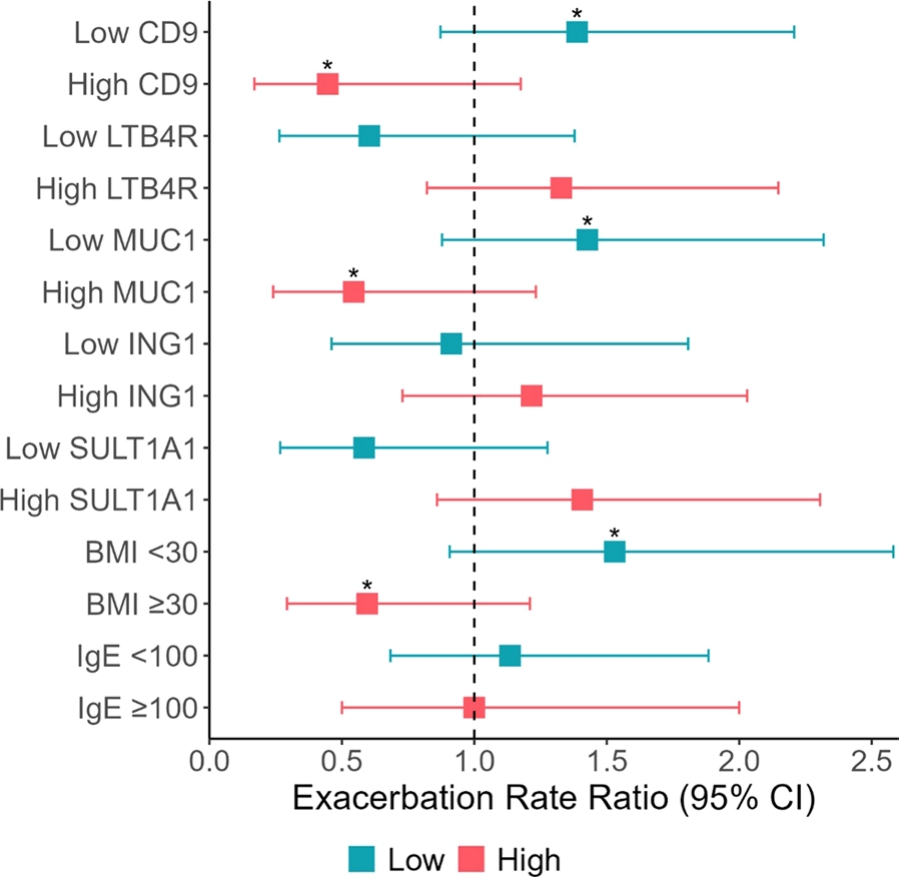
Forest plot showing the relationship between the exacerbation rate ratio and unadjusted levels of the top 5 proteins associated with omalizumab response. ‘Low’ indicates protein level below the median and ‘high’ indicates levels at or above the median. Exacerbation rate ratios < 1.0 indicate better response to mepolizumab, that is greater reductions asthma exacerbation.. *comparing groups, e.g., high vs low: p < 0.05. BMI, body mass index; CI, confidence interval; IgE, immunoglobulin E

The exacerbation rate was lower in patients with high levels of CD9 and MUC1. For SULT1A1, the exacerbation rate was higher in patients with high levels (Fig. 5). The association between IgE ≥ 100 or < 100 and the change in exacerbations was not significantly different.

GSEA demonstrated 60 significantly enriched functional groups at FDR-corrected P < 0.05 and 145 at P < 0.20 associated with response to omalizumab (top 5 gene sets represented in Additional file 1: Fig. S5; top 20 gene sets shown in Additional file 1: Table S6). The strongest associations were with intracellular transport (NES 2.02, unadjusted p = 4.63 × 10E−08, FDR-corrected: 0.0001), peptidyl-threonine phosphorylation (NES 2.08, unadjusted p = 6.49 × 10E−06, FDR-corrected: 0.0036), and phosphorus metabolic process (NES 1.52, unadjusted p = 8.43 × 10E−06, FDR-corrected: 0.0036). The network map highlighted functional groups intracellular transport, phosphorylation, phosphate-containing compound metabolic process, and cellular macromolecule metabolic processes with leading edge interactions through ING1, MAPK3, SYK, HEXIM1, DERL1, and SULT1A1. (Additional file 1: Fig. S6 and Table S6).

### PPI network of omalizumab response module

**Fig. 6:**
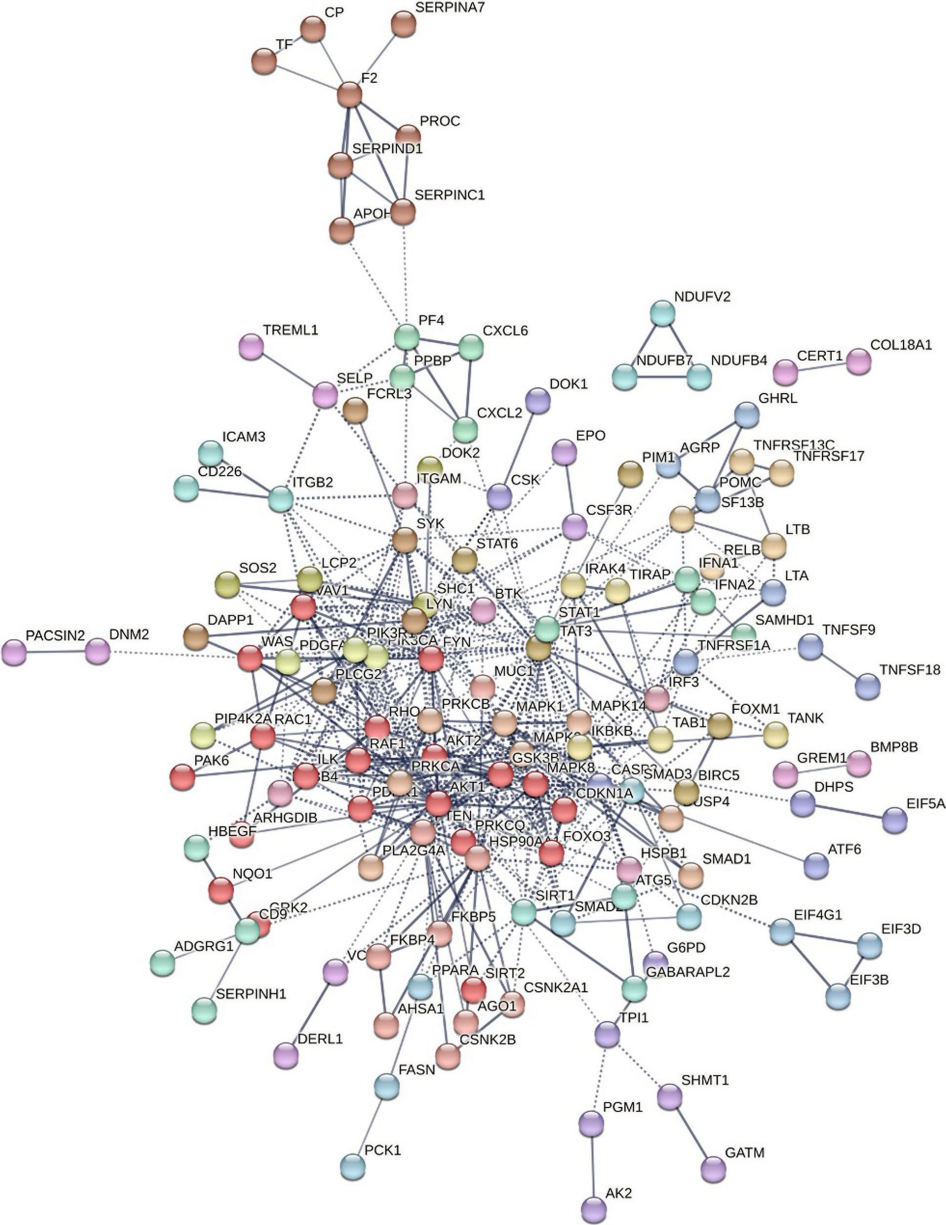
Protein–Protein Interaction (PPI) network of omalizumab response. Network including interactions with at least a score of high confidence (≥ 0.70), 10 interactors in first shell, and 5 in the second shell. Clusters (color-coded) were generated using the Markov Cluster Algorithm (MCL) embedded in the STRING database

The omalizumab response module included 428 nodes and 1864 edges with a P-value for enrichment of < 1.0 × 10E-17. Unsupervised MCL clustering generated 87 clusters with 40 of these clusters (46%) having a node degree of 3 or more, that is had at least two interactions within the network. When limited to proteins that met the FDR corrected significance threshold of < 0.05, there were 187 nodes, 204 edges, and 20 clusters with ≥ 3 proteins in the network (Fig. 6 and Additional file 1: Table S7). The top 4 clusters had 8 or more proteins. The core of Cluster 1 (20 proteins) and Cluster 4 (8 proteins) demonstrated multiple functionally enriched pathways involving protein kinases which included the AKT serine-threonine protein kinase family (AKT1 and AKT2), the Mitogen‑activated protein kinase (MAPK) signaling pathway (MAPK1, MAPK3, MAPK8, MAPK14), and the Protein Kinase C family (PRKCA and PRKCB, and PRKCQ). Cluster 2 (9 proteins) included MUC1, and two members of the casein kinase-II (CK2) class of the serine-threonine protein kinase family (CSNK2A1 and CSNK2B) (Additional file 1: Table S7). The enriched pathway with the highest strength was the Fc epsilon R1 signaling pathway (FDR P 2.88 × 10E−21) (Additional file 1: Table S8). Proteins involved in this pathway included multiple MAPK and AKT genes and the phospholipases: PLA2G4A and PLCG2.

### Overlap between mepolizumab and omalizumab

**Fig. 7:**
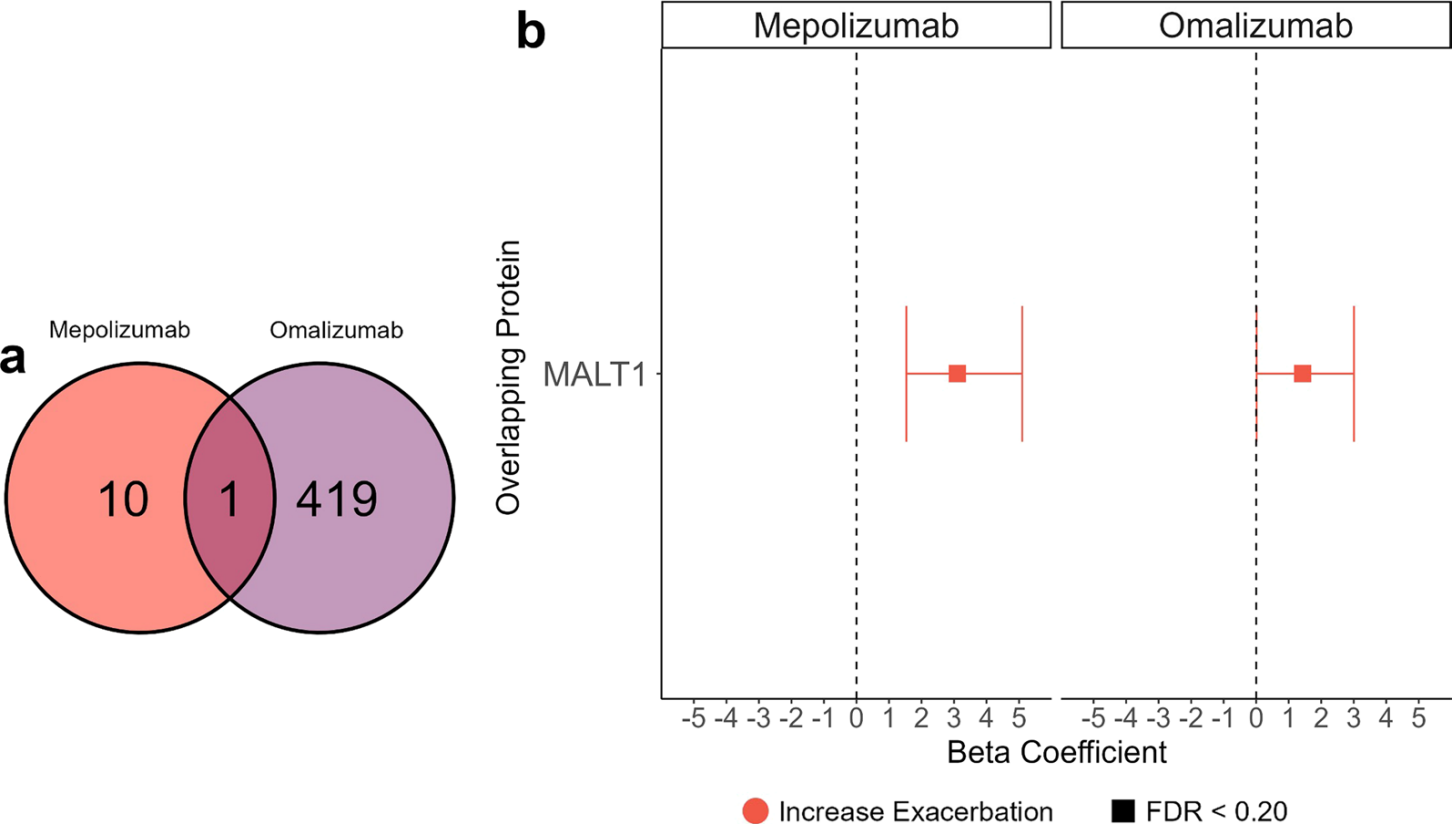
Overlapping proteins associated with the change in exacerbations in the mepolizumab group and in the omalizumab group. **a** Venn diagram indicates the numbers of significant proteins and overlapping proteins. **b** Forest plot of the singular overlapping protein (mucosa associated lymphoid tissue lymphoma translocation protein 1- MALT1) associated with change in exacerbations in both the mepolizumab and the omalizumab groups. Squares indicate proteins with significant association with FDR < 0.20. Effect sizes are presented as beta coefficients with 95% confidence intervals where positive (negative) values indicate association with increased (decreased) exacerbations following biologic initiation. The vertical dashed lines represented no difference

At the FDR-corrected p-value threshold < 0.05, no protein was associated with both omalizumab and mepolizumab response. At an FDR-corrected P < 0.20 threshold, the mucosa-associated lymphoid tissue lymphoma translocation protein was associated with both omalizumab and mepolizumab response (Fig. 7). Higher levels of MALT1 were associated with poorer response to both mepolizumab (unadjusted P 0.00047, FDR-corrected: 0.15) and omalizumab (p 0.048, FDR-corrected: 0.17). MALT1 in addition to CARD9 made up Cluster 5 in the PPI network of mepolizumab response module and was closely related to Cluster 2 which included BIRC3, TNFRSF1, and the serein/threonine-protein kinases, RIPK1 and RIPK3 (Additional file 1: Table S3). For omalizumab, MALT1 was part of the core enrichment of 11 of 52 gene sets significant at the FDR-corrected p < 0.05 level but was not part of the top 20 clusters associated with the omalizumab response module.

## Discussion

In this study, we measured 1437 proteins in plasma samples collected prior to therapy initiation from 51 patients with moderate to severe asthma who had received omalizumab or mepolizumab and sought to identify protein profiles at baseline associated with reductions in exacerbations over the twelve months of follow up. Higher levels of myostatin and LOXL2 were associated with a poor response to mepolizumab. The dichotomized levels of these proteins differentiated those with a reduction in exacerbations over follow up better than the baseline eosinophil count (≥ 300 vs < 300 cells per mcL). A higher level of LTB4R was associated with worse response to omalizumab while higher levels of CD9 antigen and MUC1 correlated with better response to omalizumab. The level of IgE, omalizumab’s target, was not significantly associated with the change in exacerbations. In GSEA, proteins associated with extracellular matrix organization were the most associated with mepolizumab response. For omalizumab, intracellular transport and metabolic processes involving phosphorylation and phosphate-containing compound processes were the most enriched. We evaluated the protein–protein interactions between the proteins associated with mepolizumab and omalizumab response to gain mechanistic insights into the functions of these proteins and the biological processes associated with response to these therapies. The TNF signaling, NF-kappa B signaling, and B cell receptor signaling pathways were enriched in the mepolizumab response module and included multiple receptor-interacting serine/threonine-protein kinases (RIPK). For omalizumab, similarly to the results of the GSEA, we found an enrichment of kinase pathways including SMAD proteins and mitogen-activated protein kinases (MAPKs), PRKC, and the CK2 protein kinase families, with the high affinity IgE receptor signaling pathway as the pathway with the strongest enrichment. To date, there has been limited information on plasma biomarkers associated with mepolizumab or omalizumab response beyond the currently available biomarkers like the peripheral eosinophil count. Thus, these results give fundamental information for potential biomarkers predictive of response to omalizumab and mepolizumab, two of the most used biologics worldwide.

Myostatin levels positively correlate with levels of the neutrophil chemoattractant, CCL20, a Th17 cells chemoattractant to inflammatory sites [27]. Glucocorticoids, a main component of treatment of persistent asthma, promote CCL20 expression in keratinocytes [28] and in the airway epithelium [29]. In the airway epithelium, CCL20 is associated with mucus hypersecretion in asthma and promotes Th17-mediated neutrophilic airway inflammation, an asthma endotype that is associated with decreased response to glucocorticoids [29, 30]. CCL20 is also higher in individuals with overweight/obese asthma and may explain the corticosteroid resistance observed in these patients [29, 31]. Our finding that higher myostatin levels are associated with poorer response to mepolizumab might imply that these patients met criteria for eosinophilic asthma based on their peripheral eosinophil counts but that the underlying mechanism driving their asthma is neutrophilic or mixed granulocytic [13, 32]. A recent study of nasal proteomics in patients within the endotype of non-eosinophilic asthma (ENDANA) study, replicated in U-BIOPRED, found that many pathways enriched in type 2 ‘T2’ asthma are also enriched in neutrophilic non-T2 asthma suggesting that there are overlapping pathways within patients of eosinophilic and neutrophilic asthma. Multiple other studies of nasal, salivary, and plasma proteomes in children and adults with asthma have shown that protein profiles differ by the presence or absence of asthma and by the degree of asthma control [13, 33, 34]. In one such study in which findings from the next gen proteomics approach was validated by enzyme-linked immunosorbent assay (ELISA), SERPINA3 was found to be higher in patients with asthma and was particularly higher in patients with an acute exacerbation of their asthma [34]. In enrichment analyses, the pathways most enriched and associated with mepolizumab response were those pertaining to the extracellular matrix organization and structure. LOXL2, which we found to be associated with poor response to mepolizumab, oxidizes lysine to allysine which crosslinks extracellular matrix (ECM) proteins [35]. A prior murine study showed that administration of the lysyl oxidase inhibitor β-aminoproprionitrile (BAPN) to TGF-β1-treated mice blocked airway collagen deposition, thereby showing the importance of this enzyme in ECM deposition, a fundamental component of airway remodeling in asthma [36].

Higher levels of CD9 antigen and MUC1 correlated with better response to omalizumab. CD9 is an extracellular vesicle (EV) associated protein demonstrable in multiple biological fluids, including the serum, plasma, urine, and airway [37, 38]. EVs are associated with many physiological processes and diseases, including lung diseases like asthma [37–39]. In a recent cohort study, EV miRNA expression profiles distinguished patients with obese type 2-low asthma from non-obese patients with type 2 low asthma [40], and in another study using the sputum lipidome from 211 patients with asthma and 41 healthy controls from U-BIOPRED [41], EV secretion by eosinophils and neutrophils was associated with increased lipid in the epithelial lining of the asthma patients. MUC1, which was also associated with better response to omalizumab, is important in allergen response as well as corticosteroid response in asthma patients [42, 43]. MUC1 knock-out mice are resistant to the anti-inflammatory effects of corticosteroids [42], and in a study of patients with asthma sensitized to pigeon, MUC1 was upregulated when these patients were exposed to the pigeon allergen [43]. Though the presence of perennial allergen sensitivity is one of the eligibility criteria for omalizumab, those with high plasma CD9 antigen and MUC1 levels may represent a subgroup of these patients that are continuously exposed to their triggers or who have clinically important perennial allergen sensitivity, and thus with better response to omalizumab. However, we found that LTB4R, also known as BLT1, was associated with worse response to omalizumab. In murine studies, blocking LTB4R led to the inhibition of both early-phases [44] and late-phases [45] of allergen-induced airway inflammation. These mixed effects warrant further investigation.

In unadjusted analyses, MALT1 was found to be associated with a poorer response to both mepolizumab and omalizumab. These effects met the FDR-corrected threshold of < 0.20 but not the stringent threshold of < 0.05. In PPI analyses, MALT1 and caspase activation and recruitment domain-9 (CARD9) formed the fifth cluster associated with the mepolizumab response module, and MALT1 was a main component of the enriched NF-kB and the B cell receptor signaling pathways. Multiple MAPKs (1, 3, 8, and 14) were associated with the omalizumab response module. CARD9 is expressed in neutrophils and antigen presenting cells including dendritic cells and macrophages and is involved in immune responses to the environment [46]. As part of the CARD-BCL10-MALT1 (CBM) complex, CARD activates the MAPK and the NF-kB pathways downstream [46], and favors a Th1/Th17 polarization [47, 48]. MAPKs are at the intersection of multiple signaling and inflammatory pathways in the airways and many studies in mice and humans have shown that MAPK inhibitors can restore corticosteroid sensitivity in patients with asthma [49, 50]. MALT1 levels, on the other hand, have been shown to be higher in children with a higher frequency of asthma exacerbations and to correlate positively with T2 asthma but negatively with T2 low asthma [51]. Taken together, if replicated in future studies, these results suggest that MALT1 or the CBM complex, may offer important clues towards the identification of a single biomarker that might reflect if an individual who meets criteria for both an anti-IL5 and anti-IgE is likely to respond to mepolizumab and omalizumab or not.

Our results should be interpreted with caution. This was a small single center study. Thus, statistical power was limited, and our results may not be generalizable to other cohorts. For instance, our population is predominantly White and might not be generalizable to more diverse populations. Our results need to be validated in larger and more diverse cohorts. However, many of the findings are biologically plausible and supported by prior asthma literature. Secondly, asthma is a heterogeneous disease and some of our findings may be related to other factors such as the underlying disease severity, other concomitant medications, and/or medication adherence. To limit this variability, we limited our cohort to patients with moderate to severe asthma, on maintenance therapy, and excluded patients with other indications which may or may not be more likely to be compliant with their medications. Third, we evaluated only the baseline levels of these proteins. However, the proteome is dynamic and longitudinal changes to the proteome might be more powerful in identifying a useful biomarker. Fourth, we did not confirm our findings with a direct immunoassay method such as ELISA and some of the associations found may be off target. Furthermore, we did not have a control group and despite our best efforts there is concern for confounding by indication in which these protein associations may be associated with the presence of asthma or not, or with asthma severity, rather than solely with therapeutic response. Nonetheless, this study lays foundational work that can be built open by larger studies and/or studies with more sophisticated study designs.

## Conclusion

In this small cohort, we found multiple proteins to be associated with mepolizumab or omalizumab response. Some of these proteins or identified pathways are associated with the extracellular matrix, some with airway response to allergens, and some with corticosteroid response or resistance. These findings warrant further research as we seek to optimize use of these biologic therapies in clinical practice.

### Supplementary Information

**Additional file 1: Figure S1.** Heat map of proteins associated with exacerbations after initiation of mepolizumab. Patients are presented on the *x*-axis by risk difference defined as the difference between the individual’s exacerbation rate in the one year after starting mepolizumab and the exacerbation rate in the year prior to mepolizumab initiation. Proteins are presented on the y-axis using hierarchical clustering with Euclidean distance and the Ward clustering based on log2-transformed and row-scaled protein expression. Heat map colors ranged from lower (purple) to higher (yellow) protein expression. **Figure S2.** Functional annotation of corresponding genes of proteins associated with the change in exacerbations on mepolizumab. The gene set enrichment analysis (GSEA) was conducted using the biological processes (BP) domain of Gene Ontology (GO). GO term with false discovery rate (FDR) < 0.20 were considered statistically significant. GSEA enrichment plot of top 4 significant gene set. The curves indicated the running cumulative enrichment score. Red (blue) line represented biological process associated with higher (lower) exacerbation. The barcode plot presented the position of genes related to the gene set. **Figure S3.** Represented a gene set (a GO term). The smaller node represented each gene with color indicating ranked metric ranged from red (higher exacerbations) to green (lower exacerbations). Edge indicated the membership of gene set. The gene set enrichment analysis (GSEA) was conducted using the biological processes (BP) domain of Gene Ontology (GO). GO term with false discovery rate (FDR) < 0.20 were considered statistically significant. **Figure S4.** Heat map of top 80 proteins associated with exacerbations after initiation of omalizumab. Patients are presented on the *x*-axis by risk difference defined as the difference between the individual’s exacerbation rate in the one year after starting omalizumab and the exacerbation rate in the year prior to omalizumab initiation. Proteins are presented on the y-axis using hierarchical clustering with Euclidean distance and the Ward clustering based on log2-transformed and row-scaled protein expression. Heat map colors ranged from lower (purple) to higher (yellow) protein expression. **Figure S5.** Functional annotation of corresponding genes of proteins associated with the change in exacerbations on omalizumab. The gene set enrichment analysis (GSEA) was conducted using the biological processes (BP) domain of Gene Ontology (GO). GO term with false discovery rate (FDR) < 0.20 were considered statistically significant. GSEA enrichment plot of top 4 significant gene sets. The curves indicated the running cumulative enrichment score. Red line represented biological process associated with higher exacerbation. The barcode plot presented the position of genes related to the gene set. **Figure S6.** Gene-Concept network plot for omalizumab group. The cnetplot presented the network of interacting genes among top 5 significant gene sets. The yellow node represented a gene set (a GO term). The smaller node represented each gene with color indicating ranked metric ranged from red (higher exacerbation) to white (lower exacerbation). Edge indicated the membership of gene set. The gene set enrichment analysis (GSEA) was conducted using the biological processes (BP) domain of Gene Ontology (GO). GO term with false discovery rate (FDR) < 0.20 were considered statistically significant. **Table S1.** Proteins associated with change in exacerbations in the mepolizumab group. **Table S2.** Enriched gene sets in the mepolizumab group using Gene ontology (GO). **Table S3.** Markov Algorithm-based Clusters associated with mepolizumab response. **Table S4.** Top KEGG pathways enriched in association with mepolizumab response. **Table S5.** Proteins associated with change in exacerbations in the omalizumab group. **Table S6.** Top 20 enriched gene sets in the omalizumab group using Gene Ontology (GO). **Table S7.** Markov Algorithm-based Clusters associated with omalizumab response. **Table S8.** Top 22 KEGG pathways enriched in association with omalizumab response.

## Data Availability

The data used for this study can be requested by collaborators following approval by the Mass General Brigham Institutional Review Board.

## Abbreviations

ECM: Extracellular matrix
EDTA: Ethylenediaminetetraacetic acid
ELISA: Enzyme-linked immunosorbent assay
EV: Extracellular vesicle
FDR: False discovery rate
GDF-8: Growth differentiation factor-8
GSEA: Gene set enrichment analyses
IRB: Institutional Review Board
LOXL2: Lysyl oxidase like protein-2
LTB4R: Leukotriene B4 receptor 1
MALT1: Mucosa-associated lymphoid tissue lymphoma translocation protein 1
MAPK: Mitogen‑activated protein kinase
MGB: Mass General Brigham
MMP-8: Neutrophil collagenase
MSTN: Myostatin
RFU: Relative fluorescent units
T2: Type 2
