# Supplementary material for: Lower myostatin and higher MUC1 levels are associated with better response to mepolizumab and omalizumab in asthma: a protein–protein interaction analyses
Source: Respir Res. 2023 Dec 6;24:305. doi: 10.1186/s12931-023-02620-1 (PMC10698971; doi:10.1186/s12931-023-02620-1)
Supplement: Supplementary file 1 — Additional file 1: Figure S1. Heat map of proteins associated with exacerbations after initiation of mepolizumab. Patients are presented on the x-axis by risk difference defined as the difference between the individual’s exacerbation rate in the one year after starting mepolizumab and the exacerbation rate in the year prior to mepolizumab initiation. Proteins are presented on the y-axis using hierarchical clustering with Euclidean distance and the Ward clustering based on log2-transformed and row-scaled protein expression. Heat map colors ranged from lower (purple) to higher (yellow) protein expression. Figure S2. Functional annotation of corresponding genes of proteins associated with the change in exacerbations on mepolizumab. The gene set enrichment analysis (GSEA) was conducted using the biological processes (BP) domain of Gene Ontology (GO). GO term with false discovery rate (FDR) < 0.20 were considered statistically significant. GSEA enrichment plot of top 4 significant gene set. The curves indicated the running cumulative enrichment score. Red (blue) line represented biological process associated with higher (lower) exacerbation. The barcode plot presented the position of genes related to the gene set. Figure S3. Represented a gene set (a GO term). The smaller node represented each gene with color indicating ranked metric ranged from red (higher exacerbations) to green (lower exacerbations). Edge indicated the membership of gene set. The gene set enrichment analysis (GSEA) was conducted using the biological processes (BP) domain of Gene Ontology (GO). GO term with false discovery rate (FDR) < 0.20 were considered statistically significant. Figure S4. Heat map of top 80 proteins associated with exacerbations after initiation of omalizumab. Patients are presented on the x-axis by risk difference defined as the difference between the individual’s exacerbation rate in the one year after starting omalizumab and the exacerbation rate in the year prior to omalizumab [file 12931_2023_2620_MOESM1_ESM.docx]

**
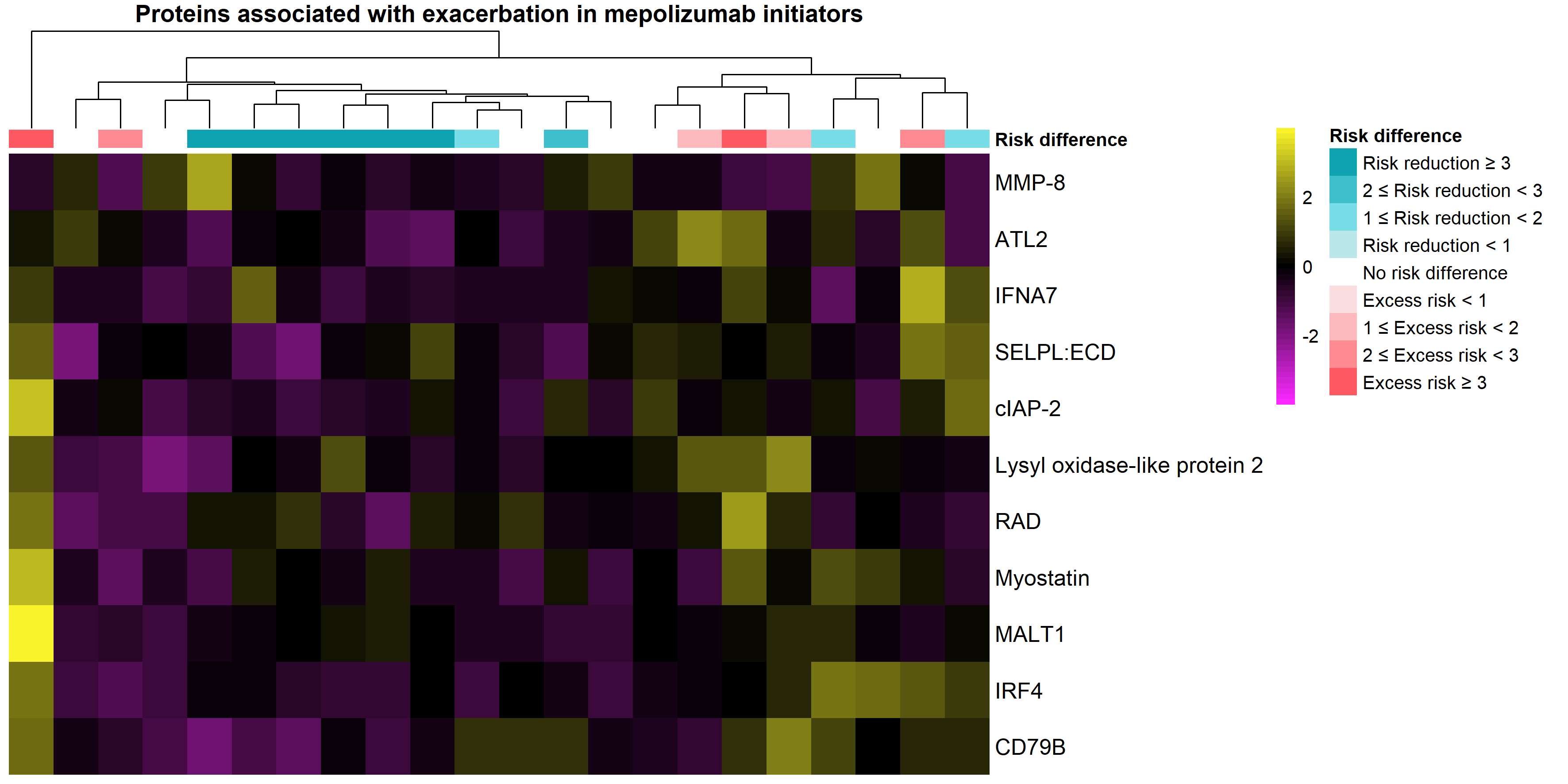
**

**Figure S1. Heat map of proteins associated with exacerbations after initiation of mepolizumab.** Patients are presented on the *x*-axis by risk difference defined as the difference between the individual’s exacerbation rate in the one year after starting mepolizumab and the exacerbation rate in the year prior to mepolizumab initiation. Proteins are presented on the y-axis using hierarchical clustering with Euclidean distance and the Ward clustering based on log2-transformed and row-scaled protein expression. Heat map colors ranged from lower (purple) to higher (yellow) protein expression.

**
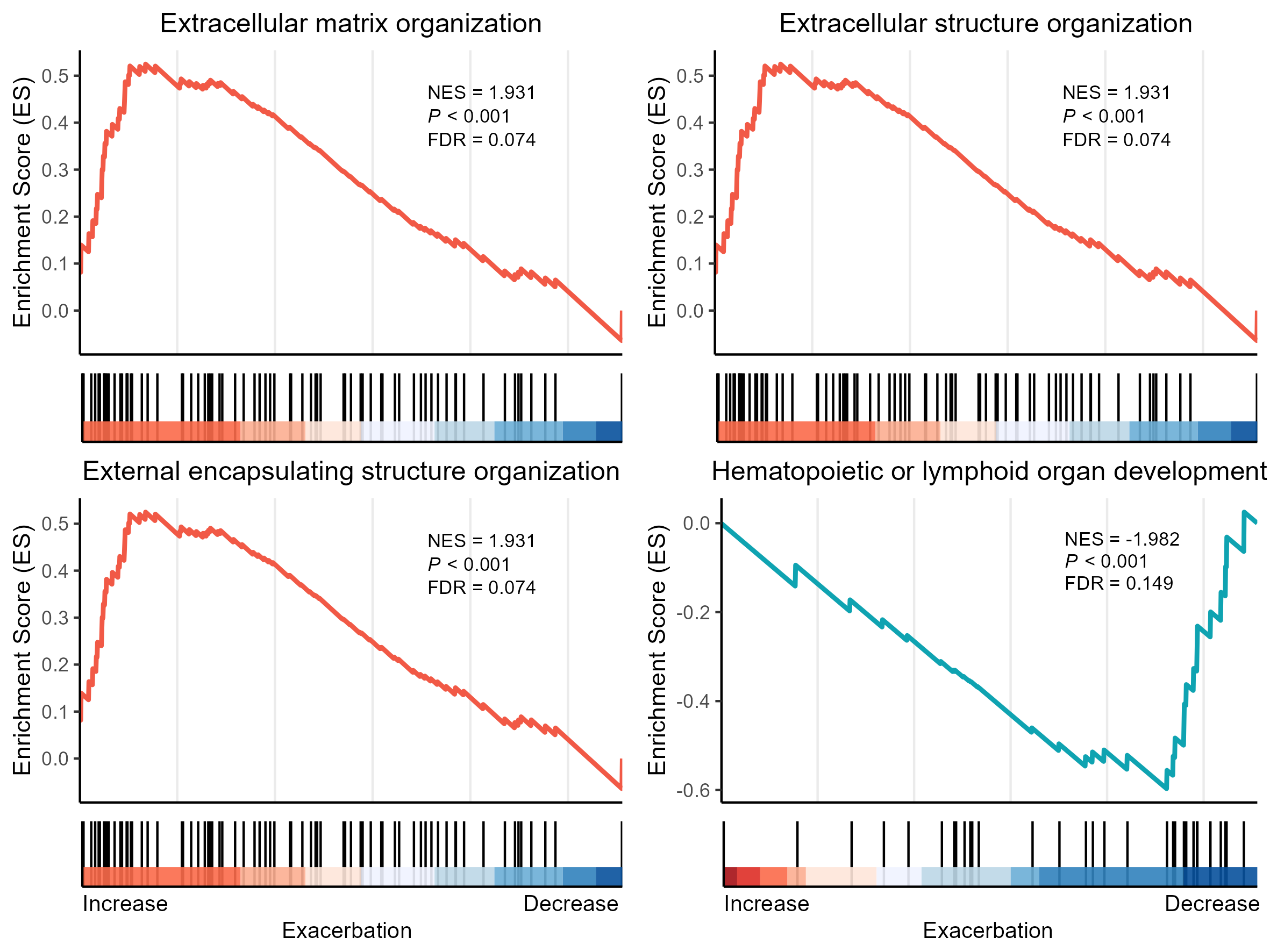
**

**Supplemental Figure E2. Functional annotation of corresponding genes of proteins associated with the change in exacerbations on mepolizumab.** The gene set enrichment analysis (GSEA) was conducted using the biological processes (BP) domain of Gene Ontology (GO). GO term with false discovery rate (FDR) < 0.20 were considered statistically significant. GSEA enrichment plot of top 4 significant gene set. The curves indicated the running cumulative enrichment score. Red (blue) line represented biological process associated with higher (lower) exacerbation. The barcode plot presented the position of genes related to the gene set.


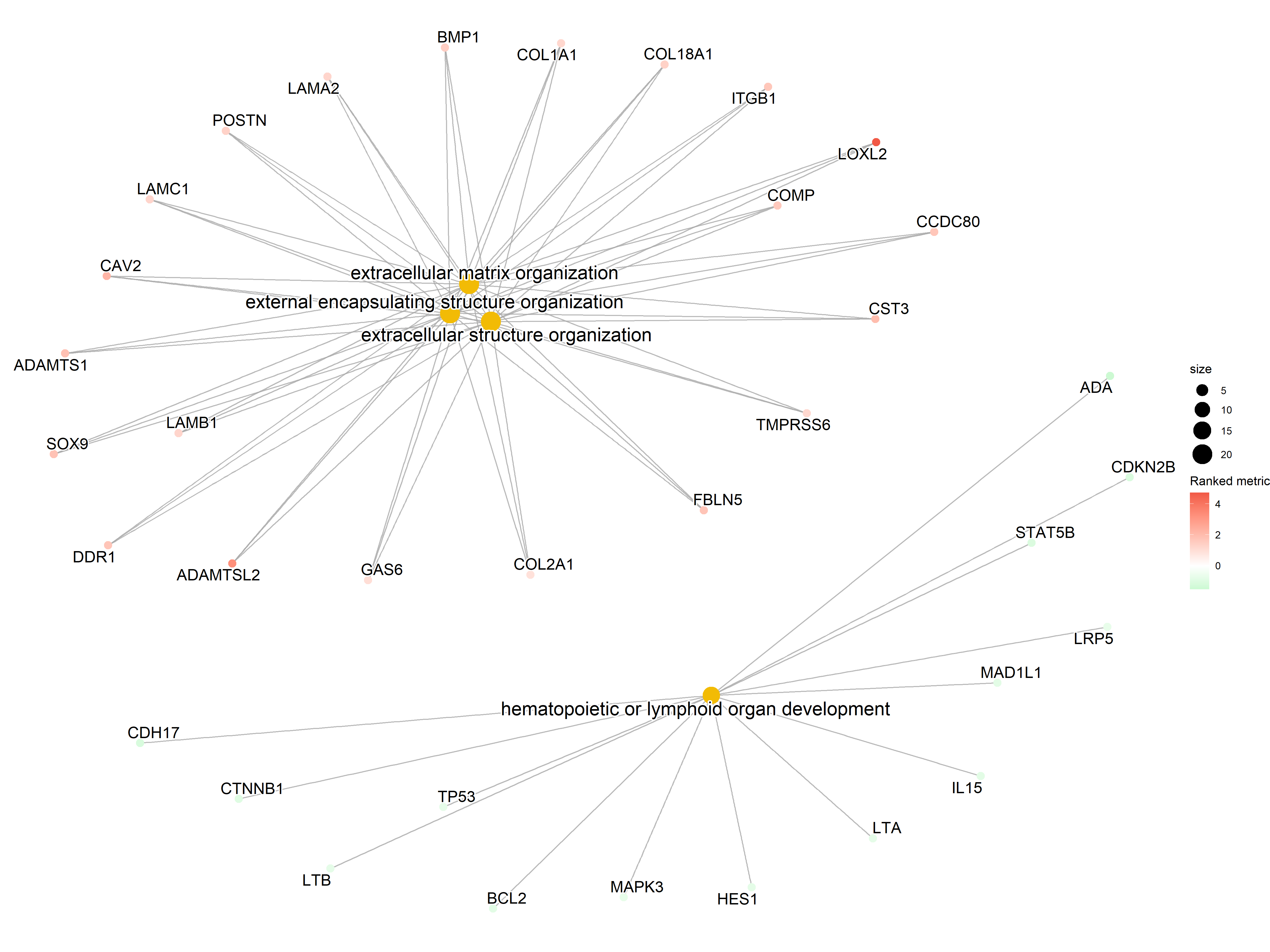


**Figure S3** represented a gene set (a GO term). The smaller node represented each gene with color indicating ranked metric ranged from red (higher exacerbations) to green (lower exacerbations). Edge indicated the membership of gene set. The gene set enrichment analysis (GSEA) was conducted using the biological processes (BP) domain of Gene Ontology (GO). GO term with false discovery rate (FDR) < 0.20 were considered statistically significant.

**
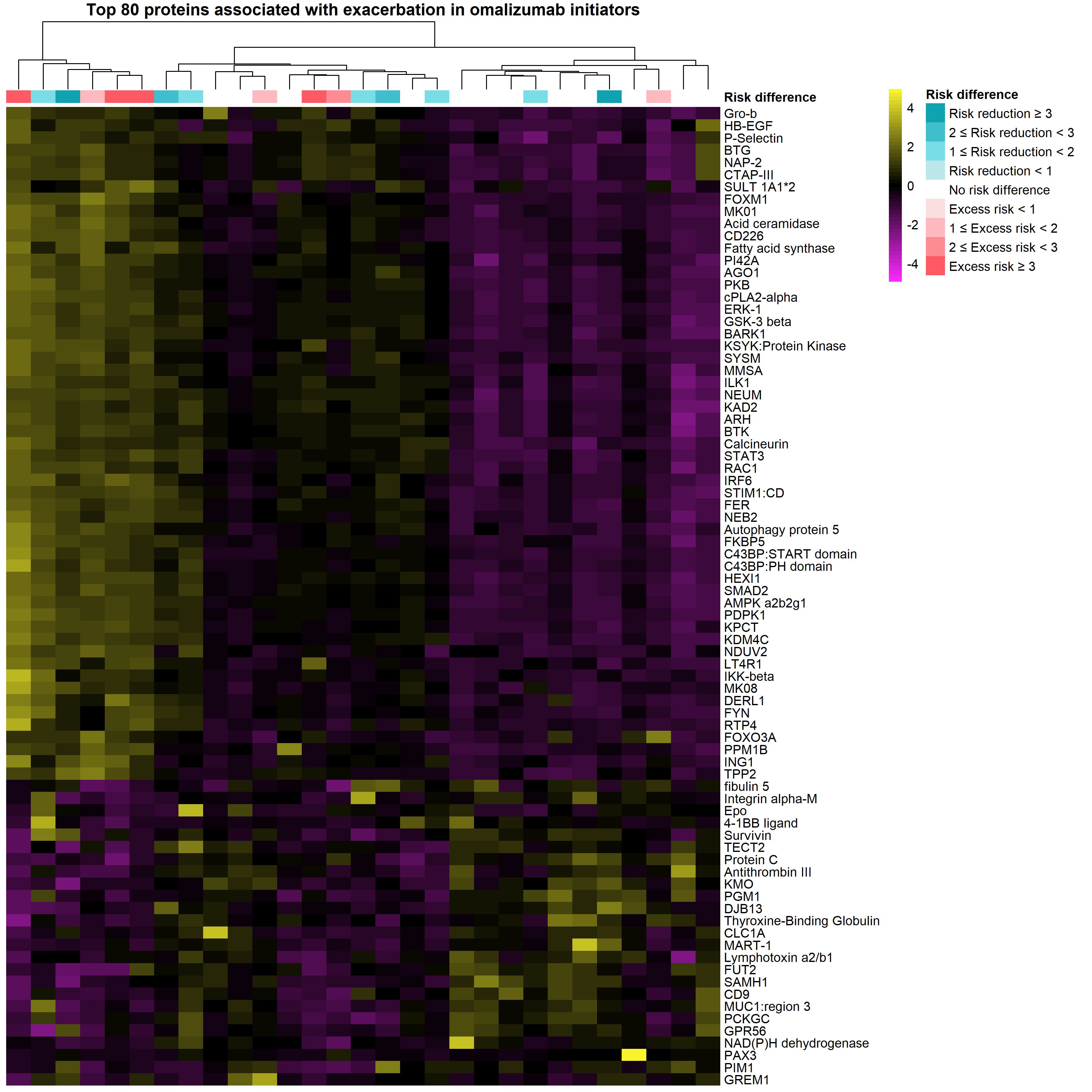
**

**Figure S4. Heat map of top 80 proteins associated with exacerbations after initiation of omalizumab.** Patients are presented on the *x*-axis by risk difference defined as the difference between the individual’s exacerbation rate in the one year after starting omalizumab and the exacerbation rate in the year prior to omalizumab initiation. Proteins are presented on the y-axis using hierarchical clustering with Euclidean distance and the Ward clustering based on log2-transformed and row-scaled protein expression. Heat map colors ranged from lower (purple) to higher (yellow) protein expression.

**
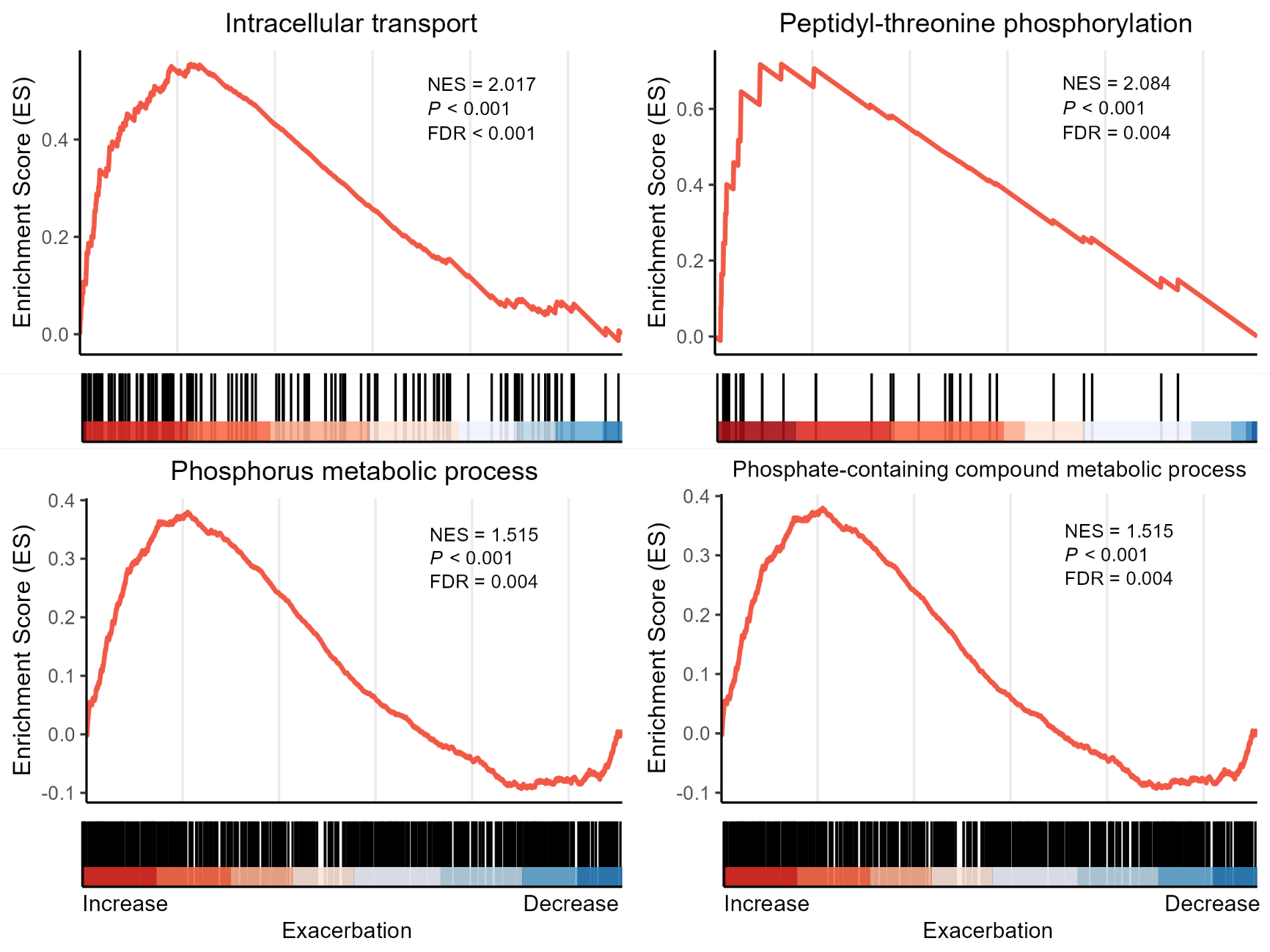
**

**Figure S5. Functional annotation of corresponding genes of proteins associated with the change in exacerbations on omalizumab.** The gene set enrichment analysis (GSEA) was conducted using the biological processes (BP) domain of Gene Ontology (GO). GO term with false discovery rate (FDR) < 0.20 were considered statistically significant. GSEA enrichment plot of top 4 significant gene sets. The curves indicated the running cumulative enrichment score. Red line represented biological process associated with higher exacerbation. The barcode plot presented the position of genes related to the gene set.

**
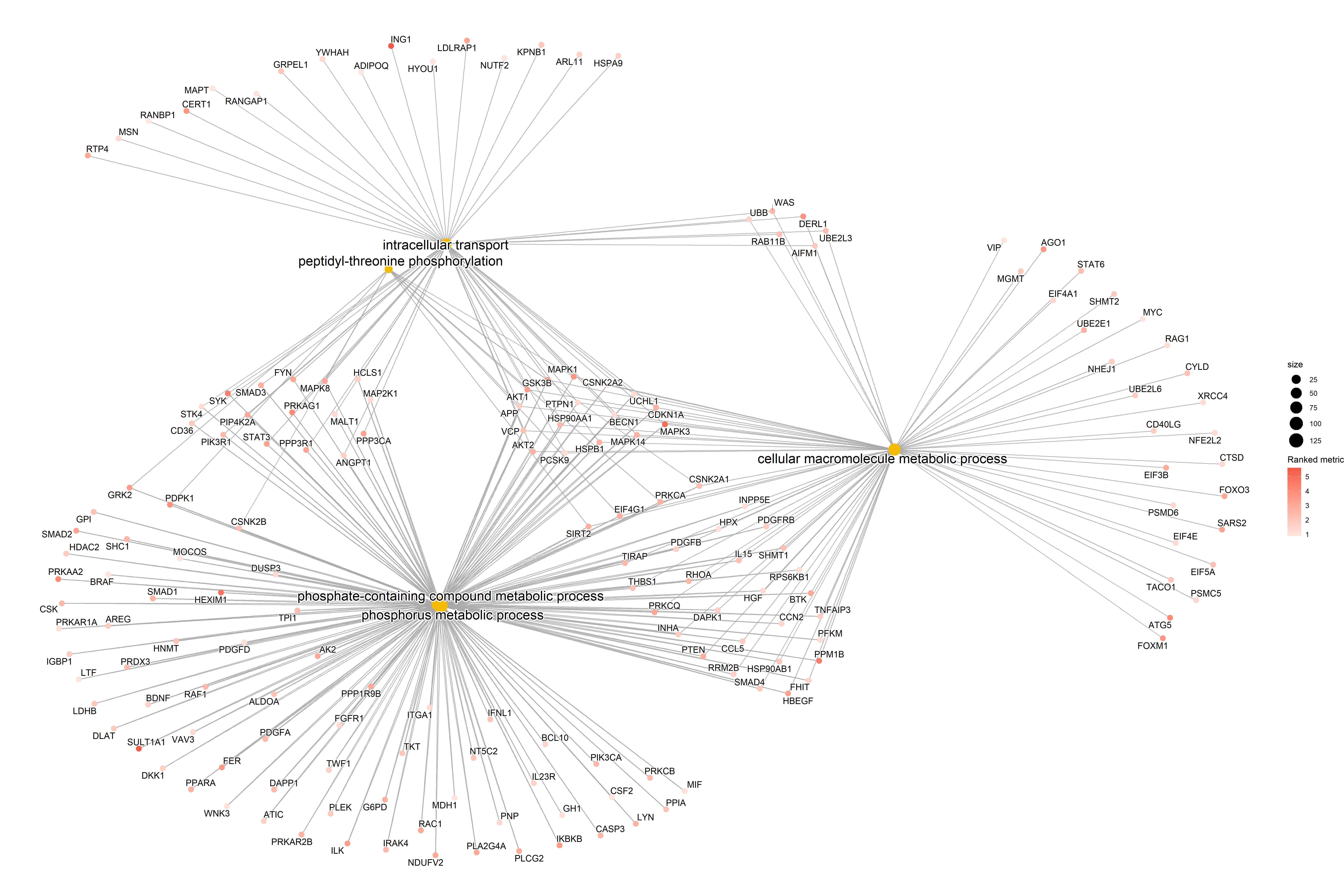
**

**Figure S6. Gene-Concept network plot for omalizumab group.** The cnetplot presented the network of interacting genes among top 5 significant gene sets. The yellow node represented a gene set (a GO term). The smaller node represented each gene with color indicating ranked metric ranged from red (higher exacerbation) to white (lower exacerbation). Edge indicated the membership of gene set. The gene set enrichment analysis (GSEA) was conducted using the biological processes (BP) domain of Gene Ontology (GO). GO term with false discovery rate (FDR) < 0.20 were considered statistically significant.

| **Table S1: Proteins associated with change in exacerbations in the mepolizumab group** | | | | | |
| --- | --- | --- | --- | --- | --- |
| **Protein** | **Abbreviation** | **EntrezGeneSymbol** | **Beta coefficient (effect size)** | **Unadjusted p-value** | **FDR corrected p-value** |
| Lysyl oxidase homolog 2 | Lysyl oxidase-like protein 2 | LOXL2 | 8.874505012 | 1.92E-05 | 0.02762285 |
| Growth/differentiation factor 8 | Myostatin | MSTN | 1.867047237 | 3.87E-05 | 0.027798692 |
| Neutrophil collagenase | MMP-8 | MMP8 | -1.657209143 | 0.00022664 | 0.108560464 |
| Mucosa-associated lymphoid tissue lymphoma translocation protein 1 | MALT1 | MALT1 | 3.104293119 | 0.00047005 | 0.145896107 |
| ADAMTS-like protein 2 | ATL2 | ADAMTSL2 | 2.285850342 | 0.00050764 | 0.145896107 |
| Interferon alpha-7 | IFNA7 | IFNA7 | 3.800027218 | 0.00078619 | 0.176043457 |
| P-selectin glycoprotein ligand 1:Extracellular domain | SELPL:ECD | SELPLG | 2.223998259 | 0.00085755 | 0.176043457 |
| GTP-binding protein RAD | RAD | RRAD | 4.228931558 | 0.00110398 | 0.182028772 |
| Interferon regulatory factor 4 | IRF4 | IRF4 | 5.188421686 | 0.00114005 | 0.182028772 |
| Baculoviral IAP repeat-containing protein 3 | cIAP-2 | BIRC3 | 4.292957884 | 0.00136928 | 0.193563619 |
| B-cell antigen receptor complex-associated protein beta chain | CD79B | CD79B | 6.83563757 | 0.0014817 | 0.193563619 |
| *Showing proteins at FDR-corrected threshold <0.20 | | | | | |

| **Table S3: Markov Algorithm-based Clusters associated with mepolizumab response** | | | | |
| --- | --- | --- | --- | --- |
| Cluster number | Cluster color | Gene count | Protein symbol | Protein name |
| 1 | Red | 6 | ACVR2B | Activin receptor type-2B |
| 1 | Red | 6 | FST | Follistatin |
| 1 | Red | 6 | FSTL3 | Follistatin-related protein 3 |
| 1 | Red | 6 | GDF11 | Growth/differentiation factor 11 |
| 1 | Red | 6 | INHBA | Inhibin beta A chain |
| 1 | Red | 6 | MSTN | Growth/differentiation factor 8 |
| 2 | Yellow | 5 | BIRC3 | Baculoviral IAP repeat-containing protein 3 |
| 2 | Yellow | 5 | RIPK1 | Receptor-interacting serine/threonine-protein kinase 1 |
| 2 | Yellow | 5 | RIPK3 | Receptor-interacting serine/threonine-protein kinase 3 |
| 2 | Yellow | 5 | TNFRSF1A | Tumor necrosis factor receptor superfamily member 1A |
| 2 | Yellow | 5 | TRAF2 | TNF receptor-associated factor 2 |
| 3 | Green | 5 | GLG1 | Golgi apparatus protein 1 |
| 3 | Green | 5 | SELE | E-selectin |
| 3 | Green | 5 | SELP | P-selectin |
| 3 | Green | 5 | SELPLG | P-selectin glycoprotein ligand 1 |
| 3 | Green | 5 | VWF | Von Willebrand antigen 2 |
| 4 | Cyan | 2 | CD79A | B-cell antigen receptor complex-associated protein alpha chain |
| 4 | Cyan | 2 | CD79B | B-cell antigen receptor complex-associated protein beta chain |
| 5 | Blue | 2 | CARD9 | Caspase recruitment domain-containing protein 9 |
| 5 | Blue | 2 | MALT1 | Mucosa-associated lymphoid tissue lymphoma translocation protein 1 |

| **Table S4: Top KEGG pathways enriched in association with mepolizumab response** | | | |
| --- | --- | --- | --- |
| Pathway | Enrichment strength | False Discovery Rate | Matching proteins in network |
| TNF signaling pathway | 1.61 | 2.69E-06 | TNFRSF1A,RIPK3,TRAF2,RIPK1,BIRC3,SELE |
| NF-kappa B signaling pathway | 1.57 | 2.06E-05 | TNFRSF1A,TRAF2,RIPK1,BIRC3,MALT1 |
| Cytosolic DNA-sensing pathway | 1.56 | 2.60E-03 | RIPK3,IFNA7,RIPK1 |
| RIG-I-like receptor signaling pathway | 1.52 | 3.20E-03 | IFNA7,TRAF2,RIPK1 |
| Necroptosis | 1.49 | 6.72E-06 | TNFRSF1A,RIPK3,IFNA7,TRAF2,RIPK1,BIRC3 |
| B cell receptor signaling pathway | 1.46 | 3.90E-03 | CD79A,CD79B,MALT1 |
| NOD-like receptor signaling pathway | 1.42 | 1.14E-05 | RIPK3,IFNA7,TRAF2,RIPK1,BIRC3,CARD9 |
| TGF-beta signaling pathway | 1.40 | 5.00E-03 | INHBA,FST,ACVR2B |
| Apoptosis | 1.36 | 1.30E-03 | TNFRSF1A,TRAF2,RIPK1,BIRC3 |
| Cell adhesion molecules | 1.34 | 1.40E-03 | GLG1,SELPLG,SELP,SELE |
| Hepatitis C | 1.29 | 2.10E-03 | TNFRSF1A,IFNA7,TRAF2,RIPK1 |
| Salmonella infection | 1.26 | 4.40E-04 | TNFRSF1A,RIPK3,TRAF2,RIPK1,BIRC3 |
| Tuberculosis | 1.26 | 2.30E-03 | TNFRSF1A,IFNA7,CARD9,MALT1 |
| Fluid shear stress and atherosclerosis | 1.25 | 1.20E-02 | TNFRSF1A,SELE,ACVR2B |
| Cytokine-cytokine receptor interaction | 1.21 | 1.10E-04 | TNFRSF1A,IFNA7,INHBA,GDF11,MSTN,ACVR2B |
| Human immunodeficiency virus 1 infection | 1.17 | 3.80E-03 | TNFRSF1A,IFNA7,TRAF2,RIPK1 |
| Human cytomegalovirus infection | 1.15 | 4.20E-03 | TNFRSF1A,IFNA7,TRAF2,RIPK1 |
| Shigellosis | 1.14 | 4.20E-03 | TNFRSF1A,TRAF2,RIPK1,MALT1 |
| Pathogenic Escherichia coli infection | 1.08 | 2.97E-02 | TNFRSF1A,TRAF2,RIPK1 |
| Kaposi sarcoma-associated herpesvirus infection | 1.08 | 2.97E-02 | TNFRSF1A,IFNA7,TRAF2 |
| Epstein-Barr virus infection | 1.07 | 2.97E-02 | IFNA7,TRAF2,RIPK1 |
| Herpes simplex virus 1 infection | 0.90 | 6.90E-03 | TNFRSF1A,IFNA7,TRAF2,BIRC3,CARD9 |
| *Showing pathways with ≥3 genes | | | |

| **Table S5: Proteins associated with change in exacerbations in the omalizumab group** | | | | | |
| --- | --- | --- | --- | --- | --- |
| **Protein** | **Abbreviation** | **EntrezGeneSymbol** | **Beta coefficient (effect size)** | **Unadjusted p-value** | **FDR corrected p-value** |
| CD9 antigen | CD9 | CD9 | -7.673417957 | 5.30E-07 | 0.00055123 |
| Leukotriene B4 receptor 1 | LT4R1 | LTB4R | 0.830803142 | 1.12E-06 | 0.00055123 |
| Mucin-1:region 3 | MUC1:region 3 | MUC1 | -7.702736085 | 1.15E-06 | 0.00055123 |
| Inhibitor of growth protein 1 | ING1 | ING1 | 2.173029819 | 2.43E-06 | 0.000873486 |
| SULT 1A1*2 | SULT 1A1*2 | SULT1A1 | 0.741795698 | 3.24E-06 | 0.000932432 |
| Antithrombin-III | Antithrombin III | SERPINC1 | -6.015151211 | 8.63E-06 | 0.001832166 |
| Mitogen-activated protein kinase 3 | ERK-1 | MAPK3 | 0.971558822 | 8.92E-06 | 0.001832166 |
| Protein HEXIM1 | HEXI1 | HEXIM1 | 0.795972598 | 1.25E-05 | 0.002242579 |
| Protein phosphatase 1B | PPM1B | PPM1B | 1.72733953 | 3.41E-05 | 0.004887492 |
| Tyrosine-protein kinase SYK:Protein kinase domain | KSYK:Protein Kinase | SYK | 1.441543959 | 3.43E-05 | 0.004887492 |
| Galactoside 2-alpha-L-fucosyltransferase 2 | FUT2 | FUT2 | -7.074006766 | 3.74E-05 | 0.004887492 |
| Phosphoglucomutase-1 | PGM1 | PGM1 | -7.738233777 | 5.87E-05 | 0.007028383 |
| Fibulin-5 | fibulin 5 | FBLN5 | -3.576980372 | 6.87E-05 | 0.007598688 |
| AMP Kinase (alpha2beta2gamma1) | AMPK a2b2g1 | PRKAA2\|PRKAB2\|PRKAG1 | 0.795148609 | 9.12E-05 | 0.00900151 |
| Autophagy protein 5 | Autophagy protein 5 | ATG5 | 1.191872253 | 9.40E-05 | 0.00900151 |
| Acid ceramidase | Acid ceramidase | ASAH1 | 1.026109477 | 0.00011445 | 0.009674584 |
| Stromal interaction molecule 1:Cytoplasmic domain | STIM1:CD | STIM1 | 0.860509641 | 0.0001274 | 0.010019592 |
| CD226 antigen | CD226 | CD226 | 0.940894352 | 0.00013248 | 0.010019592 |
| 3-phosphoinositide-dependent protein kinase 1 | PDPK1 | PDPK1 | 0.870187889 | 0.00014377 | 0.010329601 |
| MART-1 | MART-1 | MLANA | -3.256841513 | 0.00016787 | 0.011111953 |
| Derlin-1 | DERL1 | DERL1 | 0.916989125 | 0.00017092 | 0.011111953 |
| Mitogen-activated protein kinase 1 | MK01 | MAPK1 | 0.903550909 | 0.00018371 | 0.011111953 |
| Collagen type IV alpha-3-binding protein:StAR-related lipid-transfer domain, isoform 2 | C43BP:START domain | CERT1 | 0.818739455 | 0.00018559 | 0.011111953 |
| Neuromodulin | NEUM | GAP43 | 0.52044777 | 0.00019599 | 0.01126546 |
| Forkhead box protein M1 | FOXM1 | FOXM1 | 0.972411147 | 0.0002183 | 0.011359765 |
| Fatty acid synthase | Fatty acid synthase | FASN | 0.928866476 | 0.00022661 | 0.011359765 |
| CLEC-1 | CLC1A | CLEC1A | -3.486250535 | 0.000233 | 0.011359765 |
| Glycogen synthase kinase-3 beta | GSK-3 beta | GSK3B | 0.67272907 | 0.00023623 | 0.011359765 |
| Protein argonaute-1 | AGO1 | AGO1 | 0.930235247 | 0.00023716 | 0.011359765 |
| Tectonic-2 | TECT2 | TCTN2 | -5.029518566 | 0.00026466 | 0.012268432 |
| Tyrosine-protein kinase Fer | FER | FER | 0.628732084 | 0.00027692 | 0.012435368 |
| Inhibitor of nuclear factor kappa B kinase beta subunit | IKK-beta | IKBKB | 2.512661754 | 0.0002952 | 0.01285476 |
| Integrin alpha-M | Integrin alpha-M | ITGAM | -4.30666805 | 0.00031534 | 0.013327797 |
| Tripeptidyl-peptidase 2 | TPP2 | TPP2 | 1.106245463 | 0.000343 | 0.013731698 |
| Tumor necrosis factor ligand superfamily member 9 | 4-1BB ligand | TNFSF9 | -3.064812697 | 0.00036216 | 0.013731698 |
| Adhesion G-protein coupled receptor G1 | GPR56 | ADGRG1 | -3.95261016 | 0.00037173 | 0.013731698 |
| beta-adrenergic receptor kinase 1 | BARK1 | GRK2 | 0.888833481 | 0.00037176 | 0.013731698 |
| Neutrophil-activating peptide 2 | NAP-2 | PPBP | 0.619676881 | 0.00037268 | 0.013731698 |
| Beta-thromboglobulin | BTG | PPBP | 0.643325038 | 0.00039999 | 0.014332797 |
| Gro-beta | Gro-b | CXCL2 | 0.591683671 | 0.00040894 | 0.014332797 |
| RAC-alpha serine/threonine-protein kinase | PKB | AKT1 | 0.875428195 | 0.00044351 | 0.014472551 |
| FK506-binding protein 5 | FKBP5 | FKBP5 | 0.892408178 | 0.00044411 | 0.014472551 |
| Integrin-linked protein kinase | ILK1 | ILK | 0.638525261 | 0.00045274 | 0.014472551 |
| Kynurenine 3-monooxygenase | KMO | KMO | -3.223341233 | 0.00045774 | 0.014472551 |
| NAD(P)H dehydrogenase [quinone] 1 | NAD(P)H dehydrogenase | NQO1 | -1.72246178 | 0.00046328 | 0.014472551 |
| Lymphotoxin alpha2:beta1 | Lymphotoxin a2/b1 | LTA\|LTB | -2.243521997 | 0.00048177 | 0.014729974 |
| Collagen type IV alpha-3-binding protein:PH domain | C43BP:PH domain | CERT1 | 1.010829394 | 0.00049774 | 0.014901066 |
| Serine--tRNA ligase, mitochondrial | SYSM | SARS2 | 1.0330005 | 0.0005104 | 0.014968357 |
| P-selectin | P-Selectin | SELP | 1.485242502 | 0.00052655 | 0.015133172 |
| Signal transducer and activator of transcription 3 | STAT3 | STAT3 | 0.835363462 | 0.00054878 | 0.01546271 |
| Heparin-binding EGF-like growth factor | HB-EGF | HBEGF | 0.791000919 | 0.0005815 | 0.015814427 |
| Serine/threonine-protein kinase pim-1 | PIM1 | PIM1 | -3.813140958 | 0.00058327 | 0.015814427 |
| Gremlin-1 | GREM1 | GREM1 | -3.925850964 | 0.00060969 | 0.016224571 |
| Calcineurin | Calcineurin | PPP3CA\|PPP3R1 | 1.010748616 | 0.00063967 | 0.016354079 |
| Interferon regulatory factor 6 | IRF6 | IRF6 | 0.899088948 | 0.0006402 | 0.016354079 |
| Connective tissue-activating peptide III | CTAP-III | PPBP | 0.598070582 | 0.0006487 | 0.016354079 |
| Methylmalonate-semialdehyde dehydrogenase [acylating], mitochondrial | MMSA | ALDH6A1 | 0.735327297 | 0.00067156 | 0.016466546 |
| Low density lipoprotein receptor adapter protein 1 | ARH | LDLRAP1 | 0.648371284 | 0.00067608 | 0.016466546 |
| Mothers against decapentaplegic homolog 2 | SMAD2 | SMAD2 | 0.727027662 | 0.00069785 | 0.016713608 |
| Tyrosine-protein kinase Fyn | FYN | FYN | 0.853121029 | 0.00072977 | 0.017191465 |
| Forkhead box protein O3 | FOXO3A | FOXO3 | 1.514690591 | 0.00080516 | 0.01839466 |
| Protein kinase C theta type | KPCT | PRKCQ | 0.857781702 | 0.00080645 | 0.01839466 |
| NADH dehydrogenase [ubiquinone] flavoprotein 2, mitochondrial | NDUV2 | NDUFV2 | 1.966011172 | 0.00085852 | 0.018833662 |
| Vitamin K-dependent protein C | Protein C | PROC | -3.231392676 | 0.00086561 | 0.018833662 |
| Paired box protein Pax-3 | PAX3 | PAX3 | -5.368877234 | 0.00086874 | 0.018833662 |
| Tyrosine-protein kinase BTK | BTK | BTK | 0.509524933 | 0.00087812 | 0.018833662 |
| Mitogen-activated protein kinase 8 | MK08 | MAPK8 | 1.84035929 | 0.00096197 | 0.019536965 |
| Phosphoenolpyruvate carboxykinase, cytosolic [GTP] | PCKGC | PCK1 | -2.865000211 | 0.00098114 | 0.019536965 |
| Cytosolic phospholipase A2 alpha | cPLA2-alpha | PLA2G4A | 0.691713763 | 0.00098228 | 0.019536965 |
| Receptor-transporting protein 4 | RTP4 | RTP4 | 1.020973067 | 0.00098906 | 0.019536965 |
| DnaJ homolog subfamily B member 13 | DJB13 | DNAJB13 | -4.58855591 | 0.00099426 | 0.019536965 |
| Erythropoietin | Epo | EPO | -1.939167748 | 0.00101541 | 0.019536965 |
| Phosphatidylinositol 5-phosphate 4-kinase type-2 alpha | PI42A | PIP4K2A | 0.672170596 | 0.00102408 | 0.019536965 |
| Neurabin-2 | NEB2 | PPP1R9B | 0.550033476 | 0.00103707 | 0.019536965 |
| Baculoviral IAP repeat-containing protein 5 | Survivin | BIRC5 | -4.602058141 | 0.00107 | 0.019536965 |
| Thyroxine-binding globulin | Thyroxine-Binding Globulin | SERPINA7 | -3.26570488 | 0.00107017 | 0.019536965 |
| Adenylate kinase 2, mitochondrial | KAD2 | AK2 | 0.832158816 | 0.0010704 | 0.019536965 |
| Ras-related C3 botulinum toxin substrate 1 | RAC1 | RAC1 | 0.694078237 | 0.00107406 | 0.019536965 |
| Deoxynucleoside triphosphate triphosphohydrolase SAMHD1 | SAMH1 | SAMHD1 | -5.647972186 | 0.00112204 | 0.020150253 |
| Lysine-specific demethylase 4C | KDM4C | KDM4C | 0.665345846 | 0.00113582 | 0.020150253 |
| C-X-C motif chemokine 6 | GCP-2 | CXCL6 | 1.056060922 | 0.00117331 | 0.020448146 |
| Leucine-rich repeat-containing protein 59 | LRC59 | LRRC59 | 0.595846132 | 0.00119413 | 0.020448146 |
| Eukaryotic translation initiation factor 3 subunit B | EIF3B | EIF3B | 0.65262638 | 0.0011953 | 0.020448146 |
| 1-phosphatidylinositol 4,5-bisphosphate phosphodiesterase gamma-2 | PLCG2 | PLCG2 | 0.989285913 | 0.00124217 | 0.020999994 |
| Protein amnionless | AMNLS | AMN | -3.873410012 | 0.00130661 | 0.021658901 |
| NAD-dependent protein deacetylase sirtuin-2 | SIRT2 | SIRT2 | 1.293637329 | 0.00131129 | 0.021658901 |
| SH3 domain-binding glutamic acid-rich-like protein 2 | SH3L2 | SH3BGRL2 | 0.554667626 | 0.00137179 | 0.022400769 |
| Endostatin | Endostatin | COL18A1 | -3.699843726 | 0.00140852 | 0.022576072 |
| Interferon alpha-1/13 | IFNA1 | IFNA1 | -5.44192834 | 0.00141395 | 0.022576072 |
| Interferon regulatory factor 3 | IRF-3 | IRF3 | 0.747772246 | 0.00146924 | 0.023034356 |
| Glucose-6-phosphate 1-dehydrogenase | G6PD | G6PD | 0.65738925 | 0.00150002 | 0.023034356 |
| 2,4-dienoyl-CoA reductase, mitochondrial | DECR | DECR1 | 0.569171695 | 0.00150588 | 0.023034356 |
| Interferon alpha-2 | IFN-aA | IFNA2 | -4.090444267 | 0.00150677 | 0.023034356 |
| Phosphatidylinositol 3,4,5-trisphosphate 3-phosphatase and dual-specificity protein phosphatase PTEN | pTEN | PTEN | 1.139797539 | 0.00154614 | 0.023135 |
| Eukaryotic translation initiation factor 4 gamma 1 | IF4G1 | EIF4G1 | 0.695001266 | 0.0015622 | 0.023135 |
| Protein kinase C alpha type | PKC-A | PRKCA | 0.614537941 | 0.00157303 | 0.023135 |
| Cyclic AMP-dependent transcription factor ATF-6 alpha | ATF6A | ATF6 | -3.71864977 | 0.00157775 | 0.023135 |
| Mothers against decapentaplegic homolog 3 | SMAD3 | SMAD3 | 0.945383837 | 0.00163446 | 0.023724373 |
| Ubiquitin-conjugating enzyme E2 E1 | UB2E1 | UBE2E1 | 1.037738779 | 0.0016771 | 0.023976474 |
| Serine/threonine-protein kinase PAK 6 | PAK6 | PAK6 | 0.571245374 | 0.00169063 | 0.023976474 |
| Fc receptor-like protein 3 | FCRL3 | FCRL3 | -2.863568281 | 0.00173229 | 0.023976474 |
| Proto-oncogene vav | VAV | VAV1 | 0.663057236 | 0.00173244 | 0.023976474 |
| Glycine amidinotransferase, mitochondrial | GATM | GATM | 0.587351309 | 0.00174429 | 0.023976474 |
| Peroxisome proliferator-activated receptor alpha | PPARa | PPARA | 0.972412231 | 0.00175193 | 0.023976474 |
| Cyclin-dependent kinase inhibitor 1 | p21 | CDKN1A | 2.802691399 | 0.00177875 | 0.024113844 |
| Urotensin-2 | Urotensin-II | UTS2 | -2.771241127 | 0.00185649 | 0.024825091 |
| Receptor tyrosine-protein kinase erbB-4 | ERBB4 | ERBB4 | -4.061190072 | 0.00186781 | 0.024825091 |
| Mitogen-activated protein kinase 14 | MAPK14 | MAPK14 | 0.893290766 | 0.00188304 | 0.024825091 |
| Agouti-related protein | ART | AGRP | -2.777027618 | 0.00192363 | 0.025129628 |
| RAC-beta serine/threonine-protein kinase | PKB beta | AKT2 | 0.662366008 | 0.00196601 | 0.025273576 |
| Mothers against decapentaplegic homolog 1 | SMAD1 | SMAD1 | 1.790978844 | 0.00197447 | 0.025273576 |
| Serpin H1 | Collagen-binding protein | SERPINH1 | 0.627981866 | 0.00199581 | 0.025273576 |
| Prothrombin | Prothrombin | F2 | -4.156975008 | 0.002005 | 0.025273576 |
| Tyrosine-protein kinase Lyn, isoform B | LYNB | LYN | 0.67169115 | 0.00204845 | 0.025596732 |
| cAMP-dependent protein kinase type II-beta regulatory subunit | KAP3 | PRKAR2B | 0.661216658 | 0.00209519 | 0.025955074 |
| Docking protein 1 | DOK1 | DOK1 | 1.088495438 | 0.00216137 | 0.026544117 |
| Rho GDP-dissociation inhibitor 2 | Rho-GDI beta | ARHGDIB | 0.850714945 | 0.00217983 | 0.026544117 |
| Heat shock protein beta-1 | HSP 27 | HSPB1 | 0.586771828 | 0.00220912 | 0.026544117 |
| Sialate O-acetylesterase | SIAE | SIAE | 1.497020468 | 0.00221663 | 0.026544117 |
| Trem-like transcript 1 protein:Extracellular domain, Ig-like V-type domain | TRML1:ECD | TREML1 | 0.978163397 | 0.00227127 | 0.026973668 |
| SHC-transforming protein 1:Src Homology domain | SHC1:SH2 | SHC1 | 0.786633799 | 0.00247376 | 0.028928702 |
| Tyrosine-protein kinase SYK:Src Homology domain | KSYK:SH2, 1 and 2 | SYK | 0.708147767 | 0.00247615 | 0.028928702 |
| Docking protein 2 | DOK2 | DOK2 | 0.684203135 | 0.00251411 | 0.028929016 |
| Caspase-3 | Caspase-3 | CASP3 | 0.662007855 | 0.00251944 | 0.028929016 |
| Pro-opiomelanocortin | Corticotropin-lipotropin | POMC | -1.542186884 | 0.00253657 | 0.028929016 |
| Platelet-derived growth factor subunit A | PDGF-AA | PDGFA | 0.671806272 | 0.00293015 | 0.033154547 |
| Protein kinase C and casein kinase substrate in neurons protein 2 | PACN2 | PACSIN2 | 0.622036017 | 0.00297451 | 0.033393533 |
| Transforming protein RhoA | Rho A | RHOA | 0.798891846 | 0.00300953 | 0.033524814 |
| Chloride intracellular channel protein 1 | NCC27 | CLIC1 | 0.848914878 | 0.00308465 | 0.033624104 |
| Appetite-regulating hormone | ghrelin | GHRL | 0.666019606 | 0.00309027 | 0.033624104 |
| Toll/interleukin-1 receptor domain-containing adapter protein | Tirap | TIRAP | 0.928907294 | 0.00309318 | 0.033624104 |
| Intelectin-1 | Omentin | ITLN1 | -3.102241819 | 0.00311669 | 0.033624104 |
| PIK3CA/PIK3R1 | PIK3CA/PIK3R1 | PIK3CA\|PIK3R1 | 1.630300483 | 0.0031575 | 0.033624104 |
| Dynamin-2 | DYN2 | DNM2 | 0.678749629 | 0.00317259 | 0.033624104 |
| RAF proto-oncogene serine/threonine-protein kinase | c-Raf | RAF1 | 2.009780075 | 0.00321318 | 0.033624104 |
| Interleukin-1 receptor-associated kinase 4 | IRAK4 | IRAK4 | 0.958932494 | 0.00322296 | 0.033624104 |
| Wiskott-Aldrich syndrome protein | WASP | WAS | 0.817445368 | 0.00322904 | 0.033624104 |
| Prosaposin receptor GPR37 | GPR37 | GPR37 | -1.356241134 | 0.00329141 | 0.033769638 |
| Dual adapter for phosphotyrosine and 3-phosphotyrosine and 3-phosphoinositide | DAPP1 | DAPP1 | 0.7136904 | 0.00330959 | 0.033769638 |
| Thioredoxin-dependent peroxide reductase, mitochondrial | Peroxiredoxin-3 | PRDX3 | 0.640006349 | 0.00331351 | 0.033769638 |
| Chromodomain-helicase-DNA-binding protein 7 | CHD7 | CHD7 | -6.331954913 | 0.00334958 | 0.033896795 |
| Programmed cell death protein 1 | PD-1 | PDCD1 | -3.906172789 | 0.00349521 | 0.034979995 |
| Tyrosine-protein kinase CSK | CSK | CSK | 0.891339198 | 0.00352829 | 0.034979995 |
| Hsp90alpha | HSP 90a | HSP90AA1 | 0.647932548 | 0.00352964 | 0.034979995 |
| Transcription factor RelB | RELB | RELB | -3.93954735 | 0.00369395 | 0.036357586 |
| Platelet factor 4 | PF-4 | PF4 | 0.556947189 | 0.00384284 | 0.037565714 |
| Integrin beta-2 | LFA-1 beta-2 | ITGB2 | -5.59530694 | 0.00388702 | 0.037740863 |
| C-C motif chemokine 14 | HCC-1 | CCL14 | -1.241743159 | 0.00398087 | 0.038392678 |
| Dual specificity protein phosphatase 4 | DUS4 | DUSP4 | -4.129319693 | 0.00406596 | 0.038723864 |
| Bone morphogenetic protein 8B | BMP-8 | BMP8B | -2.981231725 | 0.00410575 | 0.038723864 |
| Neurogenic locus notch homolog protein 3 | Notch-3 | NOTCH3 | -2.102031186 | 0.00410947 | 0.038723864 |
| Ecto-NOX disulfide-thiol exchanger 2 | ENOX2 | ENOX2 | -5.234658771 | 0.004123 | 0.038723864 |
| Tumor necrosis factor ligand superfamily member 18 | TNFSF18 | TNFSF18 | -3.996658377 | 0.00423352 | 0.039270675 |
| Granulocyte colony-stimulating factor receptor | G-CSF-R | CSF3R | -3.514077315 | 0.00423588 | 0.039270675 |
| Triosephosphate isomerase | Triosephosphate isomerase | TPI1 | 0.721534717 | 0.00440961 | 0.04061926 |
| Protein kinase C beta type (splice variant beta-II) | PKC-B-II | PRKCB | 0.740859418 | 0.00444762 | 0.040708453 |
| Serotransferrin | Transferrin | TF | -3.377895089 | 0.00448961 | 0.040832711 |
| Aggrecan core protein | Aggrecan | ACAN | -2.384761966 | 0.00459604 | 0.041537762 |
| TRAF family member-associated NF-kappa-B activator | TANK | TANK | 0.85318651 | 0.00469306 | 0.042139744 |
| Signal transducer and activator of transcription 1-alpha/beta | STAT1 | STAT1 | 0.731010375 | 0.00476575 | 0.042139744 |
| Tumor necrosis factor ligand superfamily member 13B | BAFF | TNFSF13B | -2.207703911 | 0.00476578 | 0.042139744 |
| Signal transducer and activator of transcription 6 | STAT6 | STAT6 | 0.936316814 | 0.00477994 | 0.042139744 |
| Beta-2-glycoprotein 1 | b2-Glycoprotein I | APOH | -3.263948862 | 0.00486849 | 0.042658642 |
| Ubiquitin-conjugating enzyme E2 L3 | UB2L3 | UBE2L3 | 1.215097345 | 0.00490482 | 0.042716482 |
| Tumor necrosis factor alpha-induced protein 8 | TFIP8 | TNFAIP8 | 1.008097629 | 0.00499169 | 0.043211177 |
| Serine hydroxymethyltransferase, cytosolic | cSHMT | SHMT1 | 0.68873666 | 0.00505674 | 0.04330201 |
| Ephrin type-A receptor 7 | EPHA7 | EPHA7 | -6.279700435 | 0.00506245 | 0.04330201 |
| Ceruloplasmin | Ceruloplasmin | CP | -2.411260138 | 0.00513629 | 0.043673634 |
| Microtubule-associated serine/threonine-protein kinase 4 | MAST4 | MAST4 | -3.563608639 | 0.00521576 | 0.044088515 |
| Tumor necrosis factor receptor superfamily member 13C | BAFF Receptor | TNFRSF13C | 1.452515602 | 0.00530155 | 0.044551592 |
| Casein kinase II 2-alpha:2-beta heterotetramer | CK2-A1:B | CSNK2A1\|CSNK2B | 0.662817294 | 0.00535094 | 0.044705196 |
| Cyclin-dependent kinase 4 inhibitor B | p15-INK4b | CDKN2B | -3.218703912 | 0.00538704 | 0.044746675 |
| Differentially expressed in FDCP 6 homolog | DEFI6 | DEF6 | 1.20885764 | 0.00548514 | 0.045299677 |
| Transitional endoplasmic reticulum ATPase | TER ATPase | VCP | 0.710118829 | 0.00599394 | 0.049218842 |
| Eukaryotic translation initiation factor 5A-1 | eIF-5A-1 | EIF5A | 0.841039757 | 0.00605643 | 0.049449357 |
| *Showing proteins at FDR-corrected threshold <0.05 | | | | | |

| **Table S6: Top 20 enriched gene sets in the omalizumab group using Gene Ontology (GO)** | | | | | | | |
| --- | --- | --- | --- | --- | --- | --- | --- |
| **ID** | **Description** | **setSize** | **Enrich-**  **ment**  **Score** | **NES** | **Raw p-value** | **FDR corrected p-value** | **Core enrichment (first 10)** |
| GO:0046907 | intracellular transport | 127 | 0.555 | 2.017 | 4.63E-08 | 0.000117027 | ING1/MAPK3/SYK/PRKAG1/DERL1/MAPK1/CERT1/GSK3B/AKT1/STAT3 |
| GO:0018107 | peptidyl-threonine phosphorylation | 30 | 0.718 | 2.084 | 6.49E-06 | 0.003554465 | PDPK1/MAPK1/GSK3B/GRK2/AKT1/MAPK8/SIRT2/EIF4G1/PRKCA/CSNK2A1 |
| GO:0006793 | phosphorus metabolic process | 494 | 0.380 | 1.515 | 8.43E-06 | 0.003554465 | SULT1A1/MAPK3/HEXIM1/PPM1B/SYK/PRKAA2/PRKAG1/PDPK1/MAPK1/GSK3B |
| GO:0006796 | phosphate-containing compound metabolic process | 494 | 0.380 | 1.515 | 8.43E-06 | 0.003554465 | SULT1A1/MAPK3/HEXIM1/PPM1B/SYK/PRKAA2/PRKAG1/PDPK1/MAPK1/GSK3B |
| GO:0044260 | cellular macromolecule metabolic process | 258 | 0.423 | 1.636 | 1.51E-05 | 0.005166853 | MAPK3/PPM1B/ATG5/DERL1/MAPK1/FOXM1/GSK3B/AGO1/AKT1/SARS2 |
| GO:0060491 | regulation of cell projection assembly | 25 | 0.741 | 2.064 | 1.84E-05 | 0.005166853 | ATG5/GAP43/GSK3B/FER/RAC1/PIK3CA/PIK3R1/DNM2/WAS/HSP90AA1 |
| GO:0120032 | regulation of plasma membrane bounded cell projection assembly | 25 | 0.741 | 2.064 | 1.84E-05 | 0.005166853 | ATG5/GAP43/GSK3B/FER/RAC1/PIK3CA/PIK3R1/DNM2/WAS/HSP90AA1 |
| GO:0065009 | regulation of molecular function | 469 | 0.374 | 1.485 | 3.24E-05 | 0.008201745 | MAPK3/HEXIM1/SYK/PRKAG1/STIM1/PDPK1/DERL1/MAPK1/GSK3B/FER |
| GO:0044248 | cellular catabolic process | 240 | 0.422 | 1.618 | 4.76E-05 | 0.010736486 | MAPK3/PRKAA2/ATG5/ASAH1/DERL1/GSK3B/AGO1/AKT1/STAT3/ALDH6A1 |
| GO:1903320 | regulation of protein modification by small protein conjugation or removal | 36 | 0.662 | 1.988 | 5.29E-05 | 0.010736486 | ATG5/DERL1/AKT1/FYN/PTEN/HSP90AA1/TANK/UBE2L3/VCP/PPIA |
| GO:0051130 | positive regulation of cellular component organization | 206 | 0.424 | 1.605 | 6.78E-05 | 0.011601704 | MAPK3/SYK/MAPK1/GSK3B/FER/PPP3CA/LDLRAP1/FYN/PRKCQ/BTK |
| GO:0038093 | Fc receptor signaling pathway | 25 | 0.717 | 1.996 | 7.70E-05 | 0.011601704 | SYK/FER/IKBKB/FYN/PRKCQ/BTK/MAPK8/PLCG2/VAV1/LYN |
| GO:0006886 | intracellular protein transport | 85 | 0.536 | 1.851 | 8.18E-05 | 0.011601704 | ING1/SYK/DERL1/GSK3B/AKT1/STAT3/PPP3CA/PPP3R1/FYN/MAPK8 |
| GO:0009056 | catabolic process | 308 | 0.393 | 1.536 | 8.20E-05 | 0.011601704 | SULT1A1/MAPK3/PRKAA2/PRKAG1/ATG5/ASAH1/DERL1/GSK3B/AGO1/AKT1 |
| GO:0009894 | regulation of catabolic process | 155 | 0.446 | 1.648 | 8.39E-05 | 0.011601704 | MAPK3/PRKAA2/PRKAG1/ATG5/GSK3B/AKT1/STAT3/FYN/FOXO3/MAPK8 |
| GO:0044087 | regulation of cellular component biogenesis | 152 | 0.464 | 1.711 | 9.76E-05 | 0.012343034 | SYK/PRKAA2/ATG5/GAP43/GSK3B/FER/IKBKB/SELP/BTK/MAPK8 |
| GO:0006996 | organelle organization | 284 | 0.393 | 1.528 | 0.0001568 | 0.018031496 | MAPK3/PRKAA2/ATG5/PDPK1/MAPK1/CERT1/GAP43/GSK3B/FER/IKBKB |
| GO:0003012 | muscle system process | 77 | 0.530 | 1.811 | 0.0002202 | 0.019882757 | LTB4R/CERT1/GRK2/PPP3CA/FOXO3/G6PD/PRKCA/SMAD3/GATM/PPARA |
| GO:0072594 | establishment of protein localization to organelle | 58 | 0.562 | 1.851 | 0.000228 | 0.019882757 | ING1/SYK/AKT1/STAT3/PPP3CA/PPP3R1/MAPK8/SMAD3/CDKN1A/MAPK14 |
| GO:0033554 | cellular response to stress | 291 | 0.387 | 1.506 | 0.0002438 | 0.020224319 | MAPK3/PRKAA2/ATG5/DERL1/MAPK1/CERT1/GAP43/FOXM1/GSK3B/FER |

| **Table S7: Markov Algorithm-based Clusters associated with omalizumab response** | | | | | | | |
| --- | --- | --- | --- | --- | --- | --- | --- |
| Cluster number | Cluster color | Gene count | Protein symbol | Cluster number | Cluster color | Gene count | Protein symbol |
| 1 | Red | 20 | AKT1 | 7 | Brown | 5 | BIRC5 |
| 1 | Red | 20 | AKT2 | 7 | Brown | 5 | FOXM1 |
| 1 | Red | 20 | ARHGDIB | 7 | Brown | 5 | PIM1 |
| 1 | Red | 20 | CDKN1A | 7 | Brown | 5 | STAT3 |
| 1 | Red | 20 | FOXO3 | 7 | Brown | 5 | STAT6 |
| 1 | Red | 20 | FYN | 8 | Yellow | 5 | IKBKB |
| 1 | Red | 20 | GRK2 | 8 | Yellow | 5 | IRAK4 |
| 1 | Red | 20 | GSK3B | 8 | Yellow | 5 | TAB1 |
| 1 | Red | 20 | ILK | 8 | Yellow | 5 | TANK |
| 1 | Red | 20 | MAPK8 | 8 | Yellow | 5 | TIRAP |
| 1 | Red | 20 | NQO1 | 9 | Dark Golden Rod | 4 | DOK2 |
| 1 | Red | 20 | PAK6 | 9 | Dark Golden Rod | 4 | LCP2 |
| 1 | Red | 20 | PDPK1 | 9 | Dark Golden Rod | 4 | SHC1 |
| 1 | Red | 20 | PRKCQ | 9 | Dark Golden Rod | 4 | SOS2 |
| 1 | Red | 20 | RAC1 | 10 | Khaki | 4 | PDGFA |
| 1 | Red | 20 | RAF1 | 10 | Khaki | 4 | PIK3CA |
| 1 | Red | 20 | RHOA | 10 | Khaki | 4 | PIK3R1 |
| 1 | Red | 20 | SIRT2 | 10 | Khaki | 4 | PIP4K2A |
| 1 | Red | 20 | VAV1 | 11 | Green | 4 | CXCL2 |
| 1 | Red | 20 | WAS | 11 | Green | 4 | CXCL6 |
| 2 | Salmon | 9 | AGO1 | 11 | Green | 4 | PF4 |
| 2 | Salmon | 9 | AHSA1 | 11 | Green | 4 | PPBP |
| 2 | Salmon | 9 | CSNK2A1 | 12 | Aquamarine 3 | 4 | IFNA1 |
| 2 | Salmon | 9 | CSNK2B | 12 | Aquamarine 3 | 4 | IFNA2 |
| 2 | Salmon | 9 | FKBP4 | 12 | Aquamarine 3 | 4 | SAMHD1 |
| 2 | Salmon | 9 | FKBP5 | 12 | Aquamarine 3 | 4 | STAT1 |
| 2 | Salmon | 9 | HSP90AA1 | 13 | Aquamarine | 4 | ADGRG1 |
| 2 | Salmon | 9 | MUC1 | 13 | Aquamarine | 4 | CD9 |
| 2 | Salmon | 9 | PTEN | 13 | Aquamarine | 4 | HBEGF |
| 3 | Fire Brick | 8 | APOH | 13 | Aquamarine | 4 | SERPINH1 |
| 3 | Fire Brick | 8 | CP | 14 | Cyan | 3 | ATG5 |
| 3 | Fire Brick | 8 | F2 | 14 | Cyan | 3 | GABARAPL2 |
| 3 | Fire Brick | 8 | PROC | 14 | Cyan | 3 | SIRT1 |
| 3 | Fire Brick | 8 | SERPINA7 | 15 | Aquamarine 2 | 3 | CD226 |
| 3 | Fire Brick | 8 | SERPINC1 | 15 | Aquamarine 2 | 3 | ICAM3 |
| 3 | Fire Brick | 8 | SERPIND1 | 15 | Aquamarine 2 | 3 | ITGB2 |
| 3 | Fire Brick | 8 | TF | 16 | Sky Blue 3 | 3 | NDUFB4 |
| 4 | Sandy Brown | 8 | DUSP4 | 16 | Sky Blue 3 | 3 | NDUFB7 |
| 4 | Sandy Brown | 8 | MAPK1 | 16 | Sky Blue 3 | 3 | NDUFV2 |
| 4 | Sandy Brown | 8 | MAPK14 | 17 | Sky Blue | 3 | CDKN2B |
| 4 | Sandy Brown | 8 | MAPK3 | 17 | Sky Blue | 3 | SMAD2 |
| 4 | Sandy Brown | 8 | PLA2G4A | 17 | Sky Blue | 3 | SMAD3 |
| 4 | Sandy Brown | 8 | PRKCA | 18 | Blue | 3 | FASN |
| 4 | Sandy Brown | 8 | PRKCB | 18 | Blue | 3 | PCK1 |
| 4 | Sandy Brown | 8 | SMAD1 | 18 | Blue | 3 | PPARA |
| 5 | Dark Golden Rod 2 | 5 | DAPP1 | 19 | Sky Blue 2 | 3 | EIF3B |
| 5 | Dark Golden Rod 2 | 5 | FCRL3 | 19 | Sky Blue 2 | 3 | EIF3D |
| 5 | Dark Golden Rod 2 | 5 | LYN | 19 | Sky Blue 2 | 3 | EIF4G1 |
| 5 | Dark Golden Rod 2 | 5 | PLCG2 | 20 | Cornflower Blue 3 | 3 | AGRP |
| 5 | Dark Golden Rod 2 | 5 | SYK | 20 | Cornflower Blue 3 | 3 | GHRL |
| 6 | Sandy Brown 2 | 5 | LTB | 20 | Cornflower Blue 3 | 3 | POMC |
| 6 | Sandy Brown 2 | 5 | RELB |  |  |  |  |
| 6 | Sandy Brown 2 | 5 | TNFRSF13C |  |  |  |  |
| 6 | Sandy Brown 2 | 5 | TNFRSF17 |  |  |  |  |
| 6 | Sandy Brown 2 | 5 | TNFSF13B |  |  |  |  |

| **Table S8: Top 22 KEGG pathways enriched in association with omalizumab response** | | | |
| --- | --- | --- | --- |
| Pathway | Enrich-ment strength | False Discovery Rate | Matching proteins in network |
| Fc epsilon RI signaling pathway | 1.51 | 2.88E-20 | LCP2,MAPK1,SOS2,MAPK14,MAPK3,PIK3CA,PDPK1,FYN,PLA2G4A,SYK,AKT2,MAPK8,RAF1,PRKCA,PIK3R1,LYN,AKT1,VAV1,PLCG2,BTK |
| Endometrial cancer | 1.41 | 5.91E-14 | MAPK1,SOS2,MAPK3,PIK3CA,GSK3B,FOXO3,PDPK1,PTEN,AKT2,ILK,CDKN1A,RAF1,PIK3R1,AKT1 |
| VEGF signaling pathway | 1.39 | 6.73E-13 | MAPK1,MAPK14,HSPB1,MAPK3,PIK3CA,PLA2G4A,AKT2,RAF1,PRKCA,PIK3R1,AKT1,PLCG2,PRKCB |
| ErbB signaling pathway | 1.37 | 8.31E-17 | MAPK1,SOS2,HBEGF,MAPK3,PIK3CA,GSK3B,ERBB4,AKT2,MAPK8,CDKN1A,SHC1,RAF1,PRKCA,PIK3R1,AKT1,PAK6,PLCG2,PRKCB |
| EGFR tyrosine kinase inhibitor resistance | 1.37 | 5.84E-16 | MAPK1,SOS2,MAPK3,PIK3CA,STAT3,GSK3B,FOXO3,PDGFA,PTEN,AKT2,SHC1,RAF1,PRKCA,PIK3R1,AKT1,PLCG2,PRKCB |
| Prolactin signaling pathway | 1.37 | 2.29E-14 | MAPK1,SOS2,MAPK14,MAPK3,PIK3CA,STAT3,GSK3B,FOXO3,STAT1,AKT2,MAPK8,SHC1,RAF1,PIK3R1,AKT1 |
| Non-small cell lung cancer | 1.37 | 2.29E-14 | MAPK1,SOS2,MAPK3,PIK3CA,STAT3,FOXO3,PDPK1,AKT2,CDKN1A,RAF1,PRKCA,PIK3R1,AKT1,PLCG2,PRKCB |
| B cell receptor signaling pathway | 1.36 | 6.38E-16 | MAPK1,SOS2,MAPK3,PIK3CA,GSK3B,SYK,AKT2,RAF1,DAPP1,PIK3R1,LYN,IKBKB,AKT1,VAV1,PLCG2,BTK,PRKCB |
| Glioma | 1.35 | 3.69E-14 | MAPK1,SOS2,MAPK3,PIK3CA,PDGFA,PTEN,AKT2,CDKN1A,SHC1,RAF1,PRKCA,PIK3R1,AKT1,PLCG2,PRKCB |
| T cell receptor signaling pathway | 1.32 | 1.14E-17 | LCP2,MAPK1,SOS2,MAPK14,MAPK3,PRKCQ,PIK3CA,GSK3B,PDCD1,PDPK1,FYN,AKT2,MAPK8,RHOA,RAF1,PIK3R1,IKBKB,AKT1,PAK6,VAV1 |
| Pancreatic cancer | 1.32 | 5.27E-13 | MAPK1,SMAD2,MAPK3,PIK3CA,STAT3,SMAD3,STAT1,AKT2,MAPK8,CDKN1A,RAF1,PIK3R1,IKBKB,AKT1 |
| PD-L1 expression and PD-1 checkpoint pathway in cancer | 1.31 | 2.93E-15 | MAPK1,CSNK2A1,MAPK14,MAPK3,PRKCQ,PIK3CA,STAT3,PDCD1,STAT1,PTEN,CSNK2B,AKT2,TIRAP,RAF1,PIK3R1,IKBKB,AKT1 |
| NF-kappa B signaling pathway | 1.30 | 1.17E-16 | TNFRSF1A,TAB1,CSNK2A1,RELB,PRKCQ,TNFRSF13C,SYK,CSNK2B,TNFSF13B,TIRAP,LTA,LTB,CXCL2,LYN,IKBKB,IRAK4,PLCG2,BTK,PRKCB |
| Aldosterone-regulated sodium reabsorption | 1.30 | 5.87E-07 | MAPK1,MAPK3,PIK3CA,PDPK1,PRKCA,PIK3R1,PRKCB |
| Colorectal cancer | 1.28 | 1.87E-13 | MAPK1,SOS2,SMAD2,MAPK3,PIK3CA,CASP3,GSK3B,SMAD3,AKT2,MAPK8,CDKN1A,RHOA,RAF1,PIK3R1,AKT1 |
| Acute myeloid leukemia | 1.28 | 5.85E-11 | MAPK1,SOS2,MAPK3,PIK3CA,STAT3,PIM1,AKT2,RAF1,PIK3R1,IKBKB,AKT1,ITGAM |
| AGE-RAGE signaling pathway in diabetic complications | 1.27 | 1.02E-14 | MAPK1,MAPK14,SMAD2,MAPK3,PIK3CA,STAT3,CASP3,SMAD3,STAT1,PIM1,AKT2,MAPK8,PRKCA,PIK3R1,AKT1,PLCG2,PRKCB |
| Fc gamma R-mediated phagocytosis | 1.27 | 4.45E-14 | MAPK1,MAPK3,PIK3CA,PLA2G4A,SYK,WAS,DNM2,AKT2,RAF1,PRKCA,PIK3R1,LYN,AKT1,VAV1,PLCG2,PRKCB |
| Neurotrophin signaling pathway | 1.25 | 5.84E-16 | MAPK1,SOS2,ARHGDIB,MAPK14,MAPK3,PIK3CA,GSK3B,FOXO3,PDPK1,AKT2,MAPK8,RHOA,SHC1,RAF1,PIK3R1,IKBKB,AKT1,IRAK4,PLCG2 |
| FoxO signaling pathway | 1.24 | 2.51E-17 | GABARAPL2,SIRT1,MAPK1,SOS2,MAPK14,MAPK3,PIK3CA,STAT3,CDKN2B,PCK1,SMAD3,FOXO3,PDPK1,PTEN,AKT2,MAPK8,CDKN1A,RAF1,PIK3R1,IKBKB,AKT1 |
| Pertussis | 1.24 | 1.38E-10 | MAPK1,CXCL6,MAPK14,MAPK3,CASP3,TIRAP,MAPK8,ITGB2,RHOA,IRF3,IRAK4,ITGAM |
| Toll-like receptor signaling pathway | 1.23 | 1.62E-13 | MAPK1,TAB1,MAPK14,MAPK3,PIK3CA,IFNA1,STAT1,IFNA2,AKT2,TIRAP,MAPK8,PIK3R1,IKBKB,AKT1,IRF3,IRAK4 |
